# Low‐Coverage Whole‐Genome Analysis of Population Structure, Bottlenecks, and Selection in Indiana Bats Before and After White‐Nose Syndrome

**DOI:** 10.1111/mec.70172

**Published:** 2025-11-10

**Authors:** Robert Kwait, Evan A. Eskew, Sarah Gignoux‐Wolfsohn, Malin L. Pinsky, Maarten Vonhof, Brooke Hines, Brooke Maslo

**Affiliations:** ^1^ Department of Ecology, Evolution, and Natural Resources Rutgers The State University of New Jersey New Brunswick New Jersey USA; ^2^ Institute for Interdisciplinary Data Sciences University of Idaho Moscow Idaho USA; ^3^ Department of Biological Sciences University of Massachusetts Lowell Massachusetts USA; ^4^ Department of Ecology & Evolutionary Biology University of California Santa Cruz Santa Cruz California USA; ^5^ Department of Biological Sciences Western Michigan University Kalamazoo Michigan USA; ^6^ Burns & McDonnell Englewood Colorado USA

**Keywords:** conservation genetics, emerging infectious disease, evolutionary rescue, genome wide scan for selection, landscape genetics, lcWGS, rapid adaptation, wildlife disease

## Abstract

Conservation successes for the endangered Indiana bat ( *Myotis sodalis* ) in the early 2000s were largely reversed by white‐nose syndrome (WNS), a novel fungal disease that emerged in North America in 2006. Impacts have been variable among Indiana bat colonies leading to uncertainty regarding the full impact of WNS on this species. However, many colonies maintain negative population growth, threatening long‐term viability. Adaptive evolution could allow populations to persist despite disease, as has happened for other species; however, the evolutionary potential of Indiana bats remains unclear. Here, we perform low‐coverage whole‐genome sequencing to identify population structure, test for potential population bottlenecks, and scan for signatures of selection by comparing bat tissue samples from four states before and after WNS emergence. We found evidence of high connectivity across the Indiana bat range, but reduced gene flow to the colony from Northern New York. There was little evidence of a population bottleneck relating to WNS, suggesting disease‐driven mortality has not significantly altered demographics in this species. Similarly, we found little evidence of parallel selection occurring across the sample set. However, 3 genes contained outlier loci within every state, and several SNPs showed signs of parallel selection within subsets of locations. Finally, although non‐parallel allele frequency changes within a location are difficult to directly link to WNS, we found that groups of genes containing outlier loci in individual states were associated with immune, metabolic, and neural functions with a potential relationship to WNS pathophysiology.

## 1 Introduction

Globalisation of human trade and travel has facilitated the introduction of novel diseases to wildlife populations at unprecedented rates (Aguirre 2017; Marano et al. 2007; Wu et al. 2017). Invasive pathogens have caused mass mortality events in various wildlife taxa. For example, the fungus *Batrachochytrium dendrobatidis*, the causative agent of chytridiomycosis, has led to significant declines or extinction of multiple amphibian species (Kilpatrick et al. 2010; Scheele et al. 2019). Other similarly catastrophic disease introductions include the chytrid fungus *Batrachochytrium salamandrivorans* impacting salamanders (Grant et al. 2016; Martel et al. 2014) and myxoma virus in rabbits (Kerr et al. 2015). White‐nose syndrome (WNS), a particularly devastating disease affecting bats in North America, is caused by the invasive fungus *Pseudogymnoascus destructans* (Lorch et al. 2013). This pathogen grows on the exposed skin of bats during hibernation, causing a disruption of torpor patterns that leads to the premature expenditure of energy and water reserves, and potentially death (Cryan et al. 2010; Hoyt et al. 2021; Verant 2016; Verant et al. 2014). White‐nose syndrome has resulted in dramatic declines of multiple bat species since its arrival from Europe in 2006 (Cheng et al. 2021; Hoyt et al. 2021). One impacted species, the Indiana bat ( *Myotis sodalis* ), was already listed as federally endangered prior to WNS emergence in North America, suggesting it may be especially vulnerable to disease‐induced extirpation or extinction.

The Indiana bat is a North American hibernating species that ranges from the Midwest to the East Coast of the United States (USFW 2009). Indiana bats were listed as federally endangered in 1967 due to precipitous range‐wide declines (estimated at 57%) caused by destruction of summer roosting habitat, bioaccumulation of pesticides, and human disturbance of hibernacula (Gardner et al. 1990; Hicks and Novak 2002; O'Shea et al. 2016; Schmidt et al. 2000). However, this trend began to abate in the early 2000s, at least in some hibernacula. Indiana bat populations exhibited signs of positive population growth in census counts in 2003 continuing through 2007, suggesting the success of such conservation activities as access restrictions at hibernacula and summer habitat conservation (Currie 2002; Johnson et al. 2002; Thogmartin et al. 2012; USFW 2007, 2009). However, this optimistic trajectory was short‐lived due to the emergence of WNS.

Indiana bats are considered moderately to seriously impacted by WNS based on rates of decline and proportion of range affected (Cheng et al. 2021). WNS‐driven declines have been estimated as high as 70%–83% (Cheng et al. 2021; Hoyt et al. 2021; Thogmartin et al. 2012), with a median 10.3% annual decline across hibernacula (Thogmartin et al. 2012). In many sites, Indiana bat populations have not recovered and maintain a negative population growth rate (Hoyt et al. 2021; Maslo, Stringham, et al. 2017; Thogmartin et al. 2012). However, disease impacts are highly variable by both state and specific hibernaculum (Hoyt et al. 2021; Thogmartin et al. 2012). Several colonies even appear to be increasing over time, either due to increased survival and reproduction or immigration. The variability in Indiana bat declines along with the inherent uncertainty in census counts in bats has complicated assessment of WNS impacts. For example, a recent review found it difficult to determine the impact of WNS on Indiana bats specifically, with one model suggesting cumulative declines over 70% and another only 28% (Cheng et al. 2021). Despite this uncertainty, the combination of many sites with decreasing population trajectories, coupled with the negative average range‐wide growth rates, fuels concern over the long‐term viability of this endangered species (Hoyt et al. 2021; Maslo, Stringham, et al. 2017; Thogmartin et al. 2013).

Reversing negative growth rates may require management intervention, of which many options exist (Cheng et al. 2017; Gabriel et al. 2018; Kwait et al. 2022; Rocke et al. 2019; Turner et al. 2022). However, it is essential to determine the mechanism(s) driving the response of Indiana bats to WNS to promote management success (Maslo, Gignoux‐Wolfsohn, and Fefferman 2017). For one, it is necessary to determine how WNS has impacted Indiana bat populations because intervention may be unnecessary if the impacts of disease are not primarily responsible for declines or if declines have not been significant. Further, it is important to gauge whether a host is undergoing evolutionary rescue via rapid adaptation. Evolutionary rescue occurs when disease survival differs by genotype, resulting in a shift towards beneficial genotypes in the population and potentially a higher survival rate (Gonzalez et al. 2013; Whiteley et al. 2015). Species undergoing evolutionary rescue may return to viability without management intervention, although additional treatment could hasten recovery (Maslo, Gignoux‐Wolfsohn, and Fefferman 2017). An evolutionary response can also enable further conservation action if beneficial genomic loci are characterised.

A congener of the Indiana bat, the little brown bat ( *Myotis lucifugus* ), underwent severe declines range‐wide and is experiencing evolutionary rescue in response to WNS (Gignoux‐Wolfsohn et al. 2021). Indeed, some populations of little brown bats have returned to positive growth following initial declines (Hoyt et al. 2021; Maslo et al. 2015; Reichard et al. 2014). Accompanying this change in population trajectory, several independent studies have uncovered evidence of rapid evolution in this species via strong WNS‐induced selection (Auteri and Knowles 2020; Gignoux‐Wolfsohn et al. 2021). If Indiana bats are in the process of a similar evolutionary event, abatement of population declines may be facilitated by additional intervention strategies.

Here, we performed low‐coverage whole‐genome sequencing in search of population structure, bottlenecks, and selection in Indiana bats before and after the emergence of WNS. We looked for signs of population structure across samples and for evidence of changes in population structure between time periods. Similarly, we looked for signs of a population bottleneck prior to and after the emergence of WNS to determine if WNS had an impact on Indiana bat demographics. Using samples from hibernation sites across the Indiana bat range, we then conducted a multi‐method genome‐wide scan for selection (GWSS) to test for genomic regions or loci showing selective signatures.

## 2 Methods

### 2.1 Sample Collection

**FIGURE 1:**
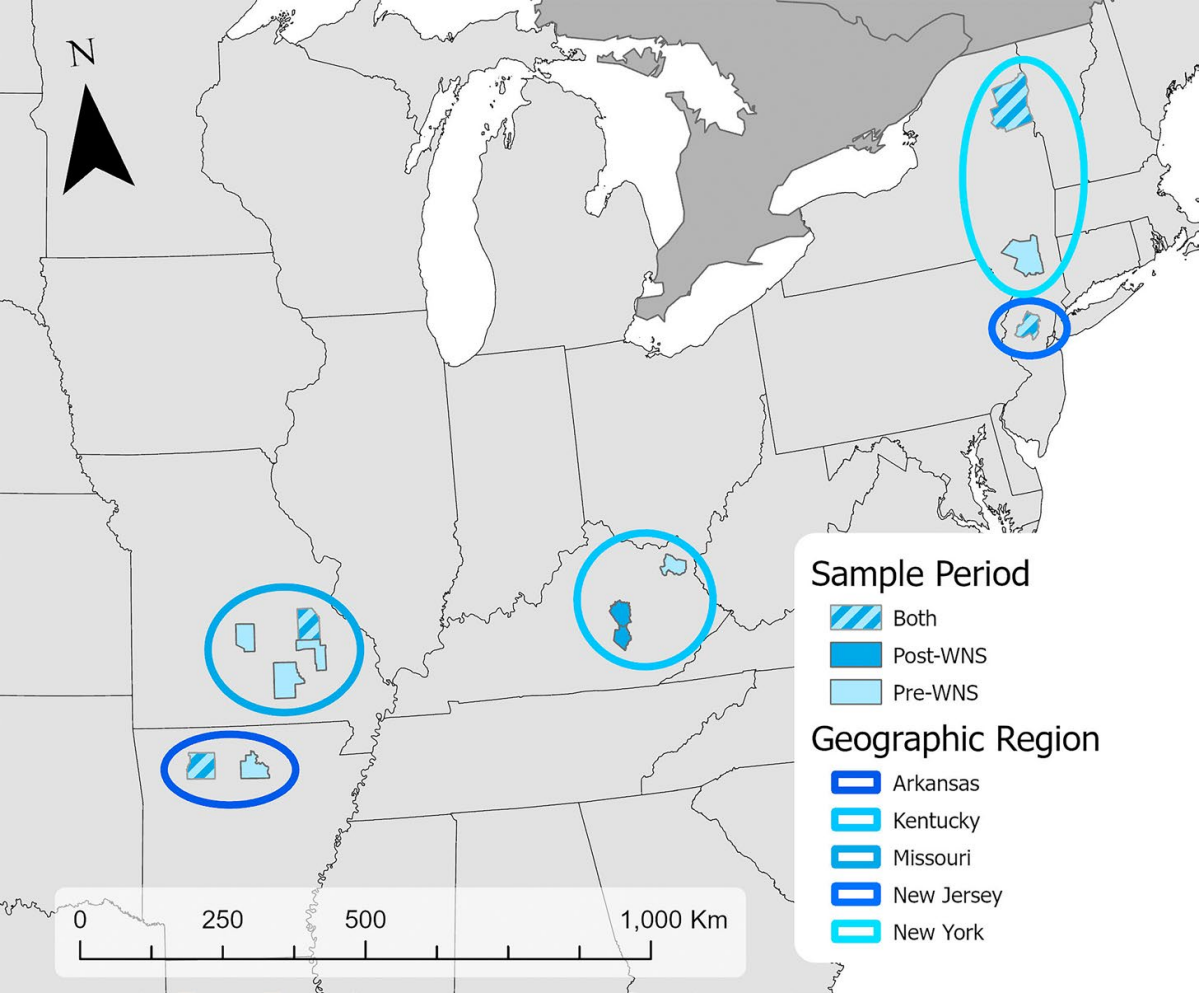
Map depicting the states from which samples were collected.

To catalogue standing genetic variation across the Indiana bat range before WNS emergence, we obtained wing tissue and DNA extract samples collected between 1998 and 2006 (*hereafter*, pre‐WNS) (Vonhof et al. 2016; Table S1). Tissue samples were preserved in dimethyl sulfoxide (DMSO), and extracted DNA was preserved in elution buffer. All samples were stored at −80°C after collection and extraction. To represent the genetic structure of Indiana bat populations persisting with WNS (*hereafter*, post‐WNS), we initially attempted to collect post‐WNS samples from the same hibernation sites where pre‐WNS samples were sourced. Due to logistical challenges (i.e., land access, site‐level extirpation), we extended our sampling footprint to include nearby hibernation sites. Therefore, for analyses, we paired pre‐ and post‐WNS samples gathered from nearby hibernacula at the scale of the state. We procured post‐WNS samples from hibernation sites within 330 km of each pre‐WNS collection site between 2017 and 2021. Samples represented five states within the Indiana bat range: Arkansas, Kentucky, Missouri, New Jersey, and New York (Figure 1).

We collected all post‐WNS wing tissue samples from torpid bats during the hibernation season. For each sample, we secured bats directly from the hibernaculum wall, gently extended one wing against a sterile plastic cutting board and used a sterile biopsy punch to take one 3‐mm tissue sample. All bats were released back into the hibernaculum immediately after processing. We placed individual samples into 1.5‐mL microcentrifuge tubes filled with RNALater (Ambion Inc., Austin, TX) and transported them to Rutgers University for storage in a −80°C freezer. All bat handling and sampling activities were performed under appropriate state and federal permits and Rutgers Institutional Animal Care and Use Committee (IACUC) protocol #999900205.

### 2.2 Library Preparation and Sequencing

We extracted genomic DNA from wing tissue samples using the QIAmp DNA Micro Kit (Qiagen, Hilden, Germany) per the manufacturer's instructions, including the use of carrier RNA to increase yield. We eluted samples with 70 μL of buffer and increased the elution incubation and centrifugation steps to 10 min each to maximise DNA yield. We then assessed the quality and concentration of extracted DNA via gel electrophoresis and the Qubit dsDNA assay (Invitrogen, Waltham, MA). An excessive number of short fragments can outcompete large fragments during PCR and disrupt fragmentation. For samples containing a high quantity of short fragments, we performed an AMPure XP bead (Beckman Coulter, Brea, CA) size selection per the manufacturer's protocol. We repeated this quality assurance approach for all samples we received as extracted DNA.

We prepared sequencing libraries using the Nextera XT kit (Illumina, San Diego, CA) following a low‐coverage whole‐genome protocol (Baym et al. 2015; Therkildsen and Palumbi 2017) modified to analyse bat‐sized genomes (Gignoux‐Wolfsohn et al. 2021). First, we fragmented and added adapters to genomic DNA using the Nextera XT tagmentation enzyme in a reduced reaction volume of 5 μL. This reaction consisted of 2.5 μL tagmentation buffer, 0.5 μL enzyme, and 2 μL DNA extract. Previous research determined that a sample concentration of 10 ng/μl achieves the ideal fragment size range of 320–550 bp for little brown bats (Gignoux‐Wolfsohn et al. 2021); however, because many of our samples had concentrations < 10 ng/μL, we diluted the enzyme to achieve the desired enzyme/DNA ratio. We then amplified tagmented DNA and added sample‐specific indexes using Illumina UD index sets A‐D. This PCR consisted of 7.52 μL of KAPA HiFi HotStart ReadyMix, 2.5 μL of Illumina indexes, and 5 μL of tagmented DNA. The cycling conditions were as follows: 72°C for 3 min, 98°C for 2.75 min, followed by 8 cycles of 98°C for 15 s, 62°C for 30 s, 72°C for 3 min, ending with a final elongation at 72°C for 1 min. Because the indexing PCR resulted in fragment sizes above the ideal 550 bp, we performed a reconditioning PCR to reduce average fragment size using primers from a previous study (Therkildsen and Palumbi 2017). This reaction consisted of 17 μL of KAPA HiFi HotStart ReadyMix and 1 μL of each reconditioning primer (1 μM) added directly to the product of the indexing PCR. Thermocycler conditions were as follows: 95°C for 5 min, 4 cycles of 95°C for 20 s, 62°C for 20 s, and 72°C for 2 min followed by a final elongation at 72°C for 2 min. We then purified resulting libraries with an AMPure XP magnetic bead cleanup following the manufacturer's protocols and assessed library quality and concentration using an Agilent TapeStation and the Qubit dsDNA assay. We then pooled libraries in equimolar ratios and sent them to Novogene (Sacramento, CA, USA) for paired‐end sequencing on 4.5 lanes of an Illumina NovaSeq 6000 with an SP flow cell and 100 nt read length.

### 2.3 Bioinformatics

Following sequencing, we demultiplexed raw reads using *barcodesplitter* (Leach and Parsons 2019) and processed them on the Amarel computer cluster and Annotate2 server at Rutgers University. For quality filtering, we followed recommendations from recent reviews on quality control for next generation and low‐coverage sequencing data (Hemstrom et al. 2024; Lou et al. 2021; O'Leary et al. 2018). We removed optical duplicates using clumpify.sh from the *bbtools* package, specifying a dupedist of 1200 as recommended for NovaSeq‐derived libraries (Bushnell 2014). We removed reads with average Phred scores < 30, 4‐bp sliding window Phred score averages < 15, low quality leading and tailing bases, adapters, and lengths < 30 bp using *TRIMMOMATIC* (Bolger et al. 2014). We then applied *fastqscreen* to remove sequences aligning to human, mouse, bacterial, fungal, or viral genomes from GenBank (Wingett and Andrews 2018). We then removed any read whose mate was removed by *fastqscreen* using *bbtools* as well as poly‐G tails (a common result of sequencing error) using *cutadapt* (Bushnell 2014; M. Martin 2011). We also filtered out reads with low variability k‐mers using *bbtools* with an entropy of 0.5, an entropy window of 50, and a k‐mer size of 5 (Bushnell 2014).

We aligned reads to the reference Myo_luc2.0 genome from ENSEMBL using the mem algorithm in the package *bwa* (Li and Durbin 2009). Default settings led to alignment errors such as erroneous matches of reads to repeat regions and large mismatches on the ends of read alignments visible in Integrated Genomics Viewer (IGV); therefore, we made the alignment more stringent by increasing the mismatch penalty to 16 (B = 16), clipping penalty to 500 (L = 500), and alignment score output minimum to 40 (T = 40). Prior to alignment, we masked repeat regions and regions with mappability scores < 1 in the reference genome using *RepeatMasker* and *GenMap* (Nishimura 2000; Pockrandt et al. 2020).

Following alignment, we transformed output SAM files into BAM files, which we then sorted and indexed using *SAMtools* (Li et al. 2009). We then removed improperly paired reads, again using *SAMtools*. We also assessed alignment quality using IGV and *SAMtools flagstat* (Thorvaldsdóttir et al. 2013). We removed duplicates and assessed genome coverage using *Picard* (http://broadinstitute.github.io/picard/). We then filtered out loci in which heterozygotes across the population had allele balance that significantly differed from 0.5 using the package *bam‐readcount* (Khanna et al. 2021) and binom.test in R to remove loci with systemic allele balance issues (Pinsky et al. 2021).

We called single nucleotide polymorphisms (SNPs) with *ANGSD*, requiring a minimum call quality score of 20, a minimum mapped quality score of 30, an SNP *p*‐value of 1 × 10^−6^, a minimum minor allele frequency (MAF) of 0.05, minimum coverage in half the individuals, and a maximum depth equal to 10× the total number of individuals for a SNP to be called (Korneliussen et al. 2014). Then we determined the percent of loci that were missing in each sample by summing the number of loci that had a likelihood of 0.33333 for each genotype in the beagle file, which represents a completely ambiguous call. We then applied exclusion thresholds of 25% and 75% missing, retaining only samples with missingness under the respective threshold. We moved through the rest of the analyses with both datasets to ensure that results were robust to the choice of missingness filter (Hemstrom et al. 2024).

We then determined specific allele frequencies within each time period for each state with one stringent and one more lenient quality filter. We ran subsequent statistics separately for each filter level and each missingness level, for a total of 4 analytical quality iterations (Hemstrom et al. 2024). We first extracted the genomic locations of variants that were called across all samples. We used the sites function in *ANGSD* to calculate MAFs in each state pre‐ and post‐WNS at the identified variant sites. We forced the major and minor alleles to match the total sample group identities and for inclusion with the more stringent filter, required a minimum quality score of 20, minimum mapping quality score of 30, a minimum number of individuals equal to half of the total individuals in each sample group, and a minimum Hardy–Weinberg deviation *p*‐value of 0.0001. For the more lenient approach, we did not impose a Hardy–Weinberg filter nor a minimum individual number. We then constructed site frequency spectra for each population using *ANGSD* with the *‐doSaf* flag so that we could identify genome‐wide trends in allele frequency distribution. After constructing site frequency spectra, we calculated average genome‐wide *F*_st_ between pre‐ and post‐WNS samples from each state.

### 2.4 Population Structure

To evaluate population structure among samples, we first performed a principal component analysis (PCA) using *PCAngsd*, which uses genotype likelihoods from variant calling to calculate eigenvectors (Meisner et al. 2021). We also used the PCA to identify and remove outlier samples because of thier potential to interfere with downstream analyses (Hemstrom et al.,  2024). We then performed admixture analysis with K‐values (number of populations) ranging from 1 to 10 (each in replicates of five) to account for each state–time period potentially being a separate population using *NGSadmix* with the *‐likes* flag to utilise genotype likelihoods (Skotte et al. 2013). We then compared log‐likelihoods and Dk (average proportion of individuals associated with a theoretical population) to determine the optimal value of *K*. We constructed PCA and admixture plots in R (Ihaka and Gentleman 1996). Lastly, we calculated average genomic differences between states by calculating pairwise *F*_st_ between all states in each time period.

### 2.5 Bottleneck Analysis

To search for signs of bottlenecks in both time periods and determine if there was a detectable bottleneck attributable to WNS, we analysed the rate of linkage disequilibrium decay, site frequency spectra and genome‐wide Tajima's D. We first calculated linkage disequilibrium and distance for each pair of SNPs across the entire pre‐WNS population and post‐WNS population using *ngsLD* setting a max distance of 10 kb (Fox et al. 2019). We compared the rate of linkage disequilibrium decay between pre‐ and post‐WNS populations, considering a reduced rate of decay in the post‐WNS population to be a sign of a bottleneck event likely attributable to WNS (Pritchard and Przeworski 2001; Slatkin 2008). We then calculated 1D folded site frequency spectra for the states in each time period using *ANGSD* with the *‐doSaf* flag and optimised them with *realSFS* (Korneliussen et al. 2014). For site frequency spectra and Tajima's D, we did not filter variants for minor allele frequency as that can bias spectra towards loci of higher allele frequency (Hemstrom et al., 2024). Site frequency spectra can demonstrate recent bottlenecks or population expansion because population size reduction leads to a loss of rare alleles and therefore a shift towards intermediate allele frequencies (Gutenkunst et al. 2009; Marth et al. 2004). Finally, we calculated genome‐wide Tajima's D in pre‐ and post‐WNS samples. Positive genome‐wide values of Tajima's D are considered a sign of a population bottleneck (Tajima 1989). To calculate genome‐wide Tajima's D, we first calculated thetas for each locus using the index file from the site frequency spectra and the *‐doThetas* flag in *ANGSD*. Then we calculated the average Tajima's D across the entire genome. We considered a positive value of genome‐wide Tajima's D to be a sign of a bottleneck and an increase in Tajima's D between pre‐ and post‐WNS populations to be a sign of a bottleneck related to WNS. We also constructed site frequency spectra and calculated genome‐wide Tajima's D for each state separately in case there were differences in population trajectories among locations.

### 2.6 Scans for Selection

We scanned the genome for signs of selective sweeps in the form of large changes in allele frequency between pre‐ and post‐WNS sample groups in each state separately. We then searched for signs of parallel selection occurring across all states. We tested at two molecular scales (genomic regions and individual SNPs) for signs of selection within each state. Because window size can influence the results of genomic region analyses (Pinsky et al. 2021), we tested two window sizes, 10,000 bp with a 2000 bp step and 50,000 bp with a 10,000 bp step (Gignoux‐Wolfsohn et al. 2021; Pinsky et al. 2021). We first used ANGSD to calculate *F*_st_ in sliding windows at both window sizes across the genome between pre‐ and post‐WNS sample groups of each state, only maintaining those with > 10 variable sites. We then employed two methods to scan for signs of a selective sweep. The first threshold‐based method scanned for genomic regions with *F*_st_ values five standard deviations greater than the genome‐wide average (Auteri and Knowles 2020; Kardos et al. 2015; Willoughby et al. 2018). For the second method, we tested for regions with higher *F*_st_ values between pre‐ and post‐WNS samples than would be expected given the distribution of such changes across the rest of the genome (Pinsky et al. 2021). This latter approach consisted of constructing a null model of *F*_st_ in each state by shuffling allele frequencies between paired loci across different positions of the genome and recalculating *F*_st_ (North et al. 2002; Pinsky et al. 2021). We performed 1000 iterations of shuffling and recalculating and generated a *p*‐value based on the actual *F*_st_ of each genomic region and the maximum expected *F*_st_ in each region from the null model (North et al. 2002).

We then scanned for genomic regions with Tajima's D values > 2 or < −2 corresponding with the timing of WNS emergence. Specifically, for each state, we considered genomic regions as candidates for selection if they had a more extreme Tajima's D value than the threshold values in the post‐WNS samples but not in the pre‐WNS samples. Tajima's D compares nucleotide diversity in genomic regions to what would be expected from neutrality in a stable population (Eckshtain‐Levi et al. 2018; Tajima 1989). A large positive value of Tajima's D, above 2 by convention, is associated with stabilizing selection or a population bottleneck (Eckshtain‐Levi et al. 2018). Conversely, a large negative value, below −2 by convention, is associated with directional selection or population growth. First, we calculated Tajima's D in sliding windows across the genome using the same two window sizes described above. For each state, we then isolated genomic windows in the post‐WNS samples with Tajima's D values > 2 or < −2 that were not above/below those values in the associated pre‐WNS samples. We also searched for individual SNPs that demonstrated large changes in allele frequency indicative of selection. For each state, we calculated the difference in minor allele frequency at each locus between pre‐ and post‐WNS samples. We considered any locus a candidate for selection if it underwent an allele frequency change five standard deviations higher than the genome‐wide average. We completed these analyses a total of 4 times, representing each combination of missingness threshold and SNP filter stringency. We considered the SNPs or genomic regions that were outliers in every iteration of quality control analysis to be the most likely to be under selection. After generating a list of genomic regions and SNPs demonstrating large changes in allele frequency, we tested for overlap between candidate loci and annotated genes by scanning each list of outlier genomic coordinates found in the previous analyses for intersection with any annotated genes from the Myo_luc2.0 genome annotation file using *bedtools* (Quinlan and Hall 2010). We consider genes to include exons, introns, and untranslated regions. In total, we had seven lists of genomic coordinates for each state corresponding with the results from each of the above analyses: *F*_st_ > 5 standard deviations above the mean in 10,000 bp and 50,000 bp windows, *F*_st_ compared to the null model in 10,000 and 50,000 bp windows, Tajima's D < −2 or > 2 in 10,000 and 50,000 bp windows, and individual SNPs that demonstrated changes in allele frequency > 5 standard deviations above the mean. We considered genes that surfaced repeatedly among genomic region and SNP based analyses within a state to be the most likely to be important and linked them to a function using the *GeneCards* database (Rebhan et al. 1998). Then, we performed a gene ontology (GO) term analysis for these genes to determine if any gene groups associated with candidate markers under selection had functional categorisations that were over‐represented in comparison to their prevalence in the genome. For this, we used the web browser program *g:profiler* (Raudvere et al. 2019), employing the GOSt function and considering a Benjamini–Hochberg adjusted *p*‐value (FDR) < 0.05 to be significant.

We then tested for evidence of parallel selection occurring on the same genetic architecture across all states. If the same loci undergo change across multiple states in conjunction with WNS emergence, there is a stronger case that WNS is the underlying cause. To investigate parallel selection, we again searched at the scale of genomic regions and individual SNPs. For parallel selection, we tested for genomic regions and SNPs that were common outliers in every state. We searched for the genomic regions that had an *F*_st_ > 5 standard deviations above the mean, a higher *F*_st_ than would be expected by chance given the null model, or values of Tajima's D outside the threshold levels in every state. Similarly, we searched for SNPs with allele frequency changes > 5 standard deviations above the mean in every state. We considered any genomic region or SNP that met the outlier threshold in every state as a candidate for parallel selection.

We also tested for windows with *F*_st_ or SNPs with allele frequency changes in the 99th percentile of magnitude in all states. We then performed a hypergeometric test using the ‘phyper()’ function in base R to determine if the number of shared windows or SNPs in the 99th percentile between states was more than would be expected if loci were drawn randomly from the genome (Pinsky et al. 2021). We counted genomic regions or SNPs as available if they were present in the dataset of each state that we used to calculate the 99th percentile. We also used the Cochran–Mantel–Haenszel (cmh) test adapted to account for genetic drift as implemented in the R package *Acer* (Spitzer et al. 2020) to scan for SNPs with large changes in allele frequency consistent with selection when considering allele changes in each state separately. The cmh test is an adapted chi‐squared test that compares allele frequencies across time points considering each population a replicate and is a relatively simple statistic that performs better than other commonly used tests (i.e., *F*_st_, diffStat) for detecting selection over two time points (Kofler and Schlötterer 2014; Vlachos et al. 2019).

The adapted cmh test requires an estimate of effective population size and the number of generations between time points to account for drift. We considered the Indiana bat generation time to be 6 years, based upon estimates of the closely related little brown bat (Gignoux‐Wolfsohn et al. 2021), and we calculated the number of generations as the average years between time points divided by the generation time (~3.21 generations). We also re‐performed the test assuming a generation time of 3 years, making the average number of generations between time points 6.42. We then used the Jorde–Ryman method of estimating effective population size from our SNP data over the two time points using code adapted from Pinsky et al. 2021 for each state. Effective population size is difficult to confidently calculate, particularly in large and widespread populations; therefore, we iteratively performed the cmh test over a range of possible effective population sizes calculated as a percentage (5%, 10%, and 20%) of the 2011 Indiana bat census population size (USFW 2019). In addition, we performed the analysis assuming an N_e_ of 1000 and 20,000 for each state. Using both methods allowed us to determine the sensitivity of the results to N_e_.

In addition to testing for selection acting on the same genomic regions or SNPs in all states, we tested for evidence of parallel selection acting on different SNPs within the same genes or on different genes with similar function across states. For all 7 analyses (*F*_st_ above threshold or greater than expected by chance compared to the null model at both window sizes, Tajima's D above threshold at both window sizes, and SNPs with above‐threshold changes in allele frequency), we looked for selection in overlapping genes across all states. We also tested for GO terms that were significantly enriched in all states even if the same genes were not significantly enriched in each state.

Next, we tested for loci likely to be under parallel selection in subsets of the states. Given variable Indiana bat survival rates across states and hibernacula, we hypothesised that parallel selection might occur in only some of the states we analysed. First, we concatenated the states into two larger geographic units based on the Indiana bat recovery units, the Northeast and Ozarks. These regions have demonstrated different overall population trends related to WNS, with the Northeast experiencing more extreme declines on average (Thogmartin et al. 2012, 2013). For the Northeast, we included all sampled hibernacula from New Jersey and New York. The remaining states were all included within the Ozarks recovery unit. We then used the cmh test, with generation time and N_e_ determined in the same manner we applied in the previous section, to search for loci that showed signs of parallel selection among states in the Northeast and Ozarks separately.

Finally, we determined the similarity between states in terms of both genomic regions and SNPs showing signs of selection. To this end, we calculated pairwise Jaccard similarity for the results of each of the 7 state‐specific scans for selection among each pair of states. In total, we created seven matrices of these pairwise similarities, one matrix for each window size of outlier values of *F*_st_, one for each window size of above threshold Tajima's D values, and one for SNPs that demonstrated above threshold allele frequency changes. We averaged the matrices in R to determine the mean Jaccard similarity for each pair of states. Then, we converted the matrix to dissimilarity and constructed a distance dendrogram in R using the *hclust* function. We considered states that exhibited the highest similarity in candidate genomic coordinates under selection across analyses most likely to be experiencing parallel selection.

## 3 Results

Overall, we obtained 340 Indiana bat samples (pre‐WNS, *N* = 173; post‐WNS, *N* = 167) and sequenced genomic DNA from 327 individuals across the five states. Sample quality was acceptable except for pre‐WNS samples sourced from Kentucky, which had low DNA concentrations and quality scores (Table S2). Therefore, we excluded Kentucky samples from the remainder of the analyses. Among the other states, sequencing yielded a total of 12,652,863,105 reads of which we retained 8,733,652,682 after filtering (Table S3). In total, we identified 5,245,285 SNPs that passed all quality filtering steps among 243 samples (Table S4). Employing missingness thresholds of 25% and 75% along with filtering PCA outliers resulted in 220 and 154 samples remaining in each dataset (Table S4). Although the missingness filter impacted the number of samples included, quality filtering did not significantly impact the number of SNPs called, with a range between 4,998,117 and 5,245,283 among all sample groups and quality iterations (Table S5).

### 3.1 Population Structure

**FIGURE 2:**
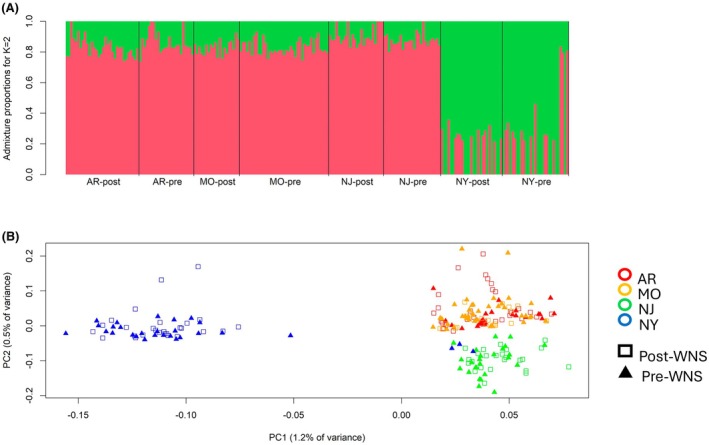
Admixture proportions plot showing the proportion of each individual's genotype associating with each of the two theoretical populations, given *K* = 2 (A). Each segment of the graph represents a time period within a state. PCA biplot of genotype likelihoods showing population separation (B). Both analyses depicted here had a missingness threshold of 75%.

Admixture analysis supported a K‐value of 1 or 2, identifying two potential populations in the dataset (Figure 2A). One of the two identified populations consisted of the northern New York samples while the rest, including the four pre‐WNS samples from southern New York, comprised the other. Likewise, the PCA demonstrated separation between samples from northern New York (Barton Hill Mine) and the rest of the Indiana bat range, although it explained a notably low amount of variance (PC1 = 1.2%, PC2 = 0.5%) (Figure 2B). Results did not differ significantly between admixture or PCA analyses employing a 25% missingness threshold and those employing a 75% missingness threshold (Figure S1). Genome‐wide *F*_st_ values between states were low (maximum *F*_st_ = 0.026) suggesting high genome‐wide similarity. The values of *F*_st_ between states depended on the missingness threshold employed. With a 75% missingness threshold, each state had the highest value of *F*_st_ with New York both pre‐ and post‐WNS, implying New York is the most differentiated in accordance with the PCA and admixture analyses (Table S6). However, at a 25% missingness threshold, the order of *F*_st_ values was more varied and changed pre‐ and post‐WNS, making inference more difficult. For example, pre‐WNS Missouri had the highest value of *F*_st_ with Arkansas (0.0226) while post‐WNS Missouri was most differentiated from New Jersey (0.0228). Together, these results suggest high gene flow across the Indiana bat range with samples gathered from northern New York state appearing more differentiated from the rest of the range despite the small geographic distances separating some hibernacula.

### 3.2 Bottleneck Analysis

**FIGURE 3:**
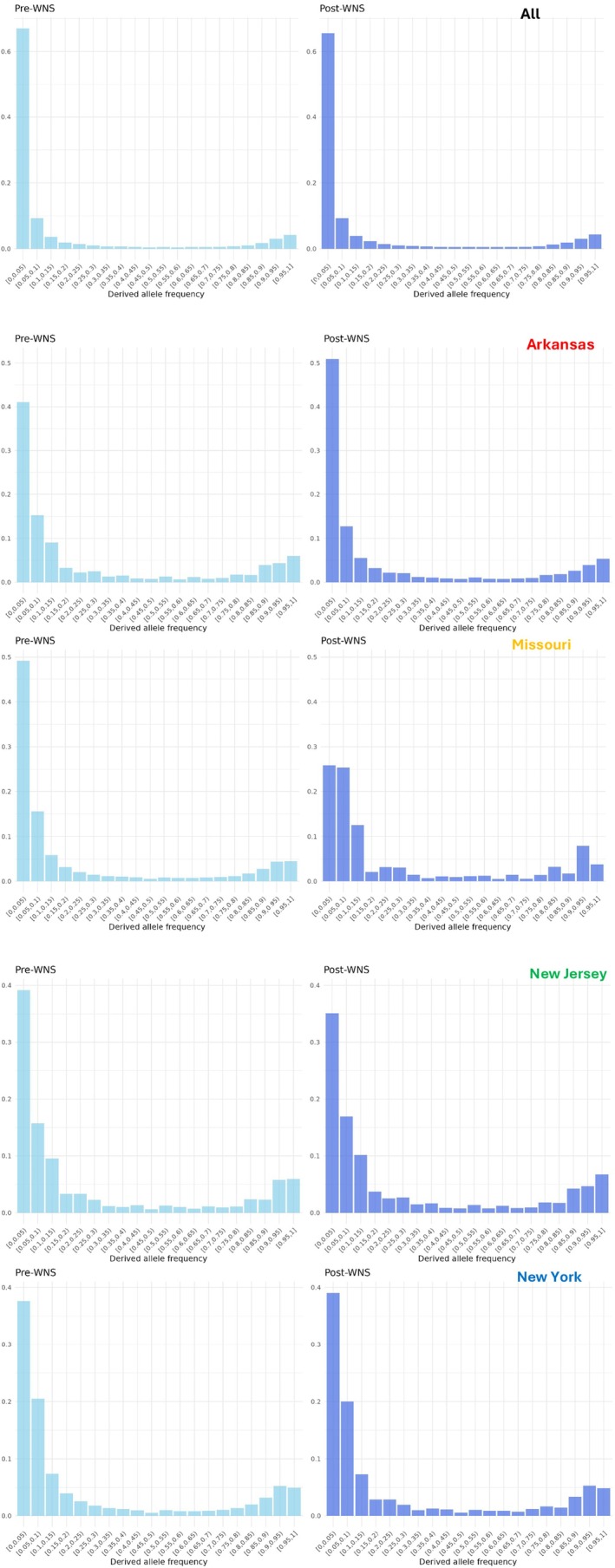
Site frequency spectrum plots for all samples in each time period. Pre‐WNS samples are on the left side and Post‐WNS samples are on the right side. The y‐axis is the frequency of occurrence and the x‐axis is the binned allele counts. These graphs were created from the data set employing a 75% missingness threshold.

Linkage disequilibrium decayed rapidly in both pre‐ and post‐WNS sample groups, passing below an *r* ^2^ of 0.2 within the 1–500 bp bin (Figure S2). Site frequency spectra demonstrated an excess of rare alleles, suggesting potential population expansion both before and after the emergence of WNS (Figure 3). Average genome‐wide Tajima's D was also negative in both time periods, supporting an excess of rare alleles. Although, Tajima's D was slightly higher in post‐WNS (−0.93) compared to pre‐WNS samples (−0.99), suggesting a relative deficit of rare alleles after the emergence of WNS (Table 1).

**TABLE 1:** Genome‐wide values of Tajima's D and *F*_st_ between pre‐ and post‐WNS samples.

State	Missingness	Pre‐WNS D	Post‐WNS D	*F*_st_
All	75	−0.992 [−0.999, −0.985]	−0.926 [−0.933, −0.919]	0.003
AR	75	−0.583 [−0.589, −0.577]	−0.690 [−0.696, −0.684]	0.011
MO	75	−0.736 [−0.742, −0.731]	−0.514 [−0.520, −0.508]	0.011
NJ	75	−0.568 [−0.575, −0.562]	−0.451 [−0.457, −0.445]	0.012
NY	75	−0.587 [−0.593, −0.581]	−0.566 [−0.572, −0.560]	0.010
All	25	−0.907 [−0.914, −0.900]	−0.872 [−0.879, −0.865]	0.004
AR	25	−0.330 [−0.334, −0.326]	−0.627 [−0.632, −0.622]	0.023
MO	25	−0.642 [−0.647, −0.637]	−0.464 [−0.469, −0.459]	0.015
NJ	25	−0.528 [−0.533, −0.523]	−0.351 [−0.355, −0.346]	0.017
NY	25	−0.567 [−0.574, −0.559]	−0.541 [−0.546, −0.536]	0.012

*Note:* All refers to the calculations for all states grouped together. Pre‐ and post‐WNS D columns refer to the values of Tajima's D. Tajima's D is presented as the mean with 95% confidence intervals. Missingness shows the maximum threshold of percent missing loci for inclusion.

In each state, we observed similar trends, with both the site frequency spectrum and genome‐wide Tajima's D implying an excess of rare alleles in almost every location (Table 1). However, the post‐WNS site frequency spectra of Missouri and New Jersey demonstrated a deficit of rare alleles relative to the associated pre‐WNS sample groups (Figure 3). Similarly, both sites demonstrated large increases in Tajima's D after the advent of WNS, supporting population contraction (Table 1). In contrast, the site frequency spectra of Arkansas showed an increase in rare alleles and a decrease in Tajima's D in the post‐WNS sample group, implying potential population expansion. New York showed very slight increases in Tajima's D in post‐WNS samples which suggests WNS did not impose a significant demographic change. These results did not change appreciably between the analyses employing a 75% or 25% missingness threshold (Figure S3).

### 3.3 Scans for Selection State

Genome‐wide *F*_st_ was low between the time periods within each state and among all samples (Table 1). The magnitude of *F*_st_ between time periods was very similar among locations, ranging from 0.010 in Arkansas to 0.012 in New Jersey with the 75% missingness threshold and from 0.012 in New York to 0.023 in Arkansas with the 25% missingness threshold. This suggests that, across all states, genome‐wide differences between time periods were similar. Within each state, multiple genomic regions had *F*_st_ values greater than 5 standard deviations from the average at both window sizes in every quality iteration of the analysis (Table 2). However, comparison to the null model of *F*_st_ returned no genomic regions with FDR < 0.05. Two states (Arkansas and New York) had genomic windows with Tajima's D > 2 in post‐WNS samples that were not shared with their pre‐WNS pair in every quality iteration (Table 2). No state had windows with values lower than −2. Similarly, SNPs in each state demonstrated allele frequency shifts > 5 standard deviations between pre‐ and post‐WNS samples in all quality iterations. The number of SNPs with large allele frequency changes ranged from 1056 in Arkansas to 4141 in New York (Table 3).

**TABLE 2:** Number of windows with *F*_st_ > 5 standard deviations of the genome‐wide average and the number of windows with Tajima's D > 2 in the post‐ but not pre‐WNS sample group for each state.

State	Missingness	Window size	Outlier *F*_st_	Outlier D
AR	75	10 k	761	721
MO	75	10 k	754	0
NJ	75	10 k	717	48
NY	75	10 k	804	161
AR	75	50 k	275	135
MO	75	50 k	213	0
NJ	75	50 k	217	3
NY	75	50 k	290	23
AR	25	10 k	694	185
MO	25	10 k	668	0
NJ	25	10 k	680	0
NY	25	10 k	775	58
AR	25	50 k	207	35
MO	25	50 k	260	0
NJ	25	50 k	223	0
NY	25	50 k	268	11
AR	Both	10 k	161	50
MO	Both	10 k	194	0
NJ	Both	10 k	184	0
NY	Both	10 k	499	10
AR	Both	50 k	58	25
MO	Both	50 k	66	0
NJ	Both	50 k	90	0
NY	Both	50 k	187	8

*Note:* Missingness demonstrates whether the analysis was done with a 75% or 25% maximum threshold. Window size shows whether the analysis used 10,000 bp windows with a 2000 bp step or 50,000 bp windows with a 10,000 bp step. Both in the missingness columns indicates the number of outlier windows that came up in both quality iterations (25% and 75% missingness).

**TABLE 3:** SNPs with allele frequency changes > 5 standard deviations above the genome‐wide average in each state with every quality iteration.

State	Missingness	Stringency	SNP increase	SNP decrease	Total
AR	75	Lenient	2683	4460	7143
MO	75	Lenient	5012	2436	7448
NJ	75	Lenient	4079	3442	7521
NY	75	Lenient	3357	3325	6682
AR	75	Stringent	2523	4054	6577
MO	75	Stringent	4056	2124	6180
NJ	75	Stringent	3415	3094	6509
NY	75	Stringent	3345	3303	6648
AR	25	Lenient	1294	6771	8065
MO	25	Lenient	5147	2249	7396
NJ	25	Lenient	5393	2479	7872
NY	25	Lenient	3405	3199	6604
AR	25	Stringent	1242	5399	6641
MO	25	Stringent	5070	2241	7311
NJ	25	Stringent	4928	2401	7329
NY	25	Stringent	3400	3197	6597
AR	Both	Both	298	758	1056
MO	Both	Both	988	557	1545
NJ	Both	Both	878	627	1505
NY	Both	Both	2085	2056	4141

*Note:* Missingness shows whether a 75% or 25% maximum missingness threshold was applied. Stringency refers to whether a more stringent (‐minInd 4, ‐HWE 0.0001) or more lenient filter was applied during the SNP call. Both in either of those sections refers to the number of SNPs that were present as outliers in every quality iteration.

Every state possessed outlier SNPs or genomic regions that overlapped genes and appeared in all quality iterations. The number of genes overlapping with candidate genomic coordinates was highest among SNPs, with the most extreme value in New York (1045). High Tajima's D windows had the lowest amount of overlap with genes, likely because of the low number of outlier windows. Within each state, at least a few genes contained *F*_st_ outlier genomic regions and SNPs in every threshold‐based analysis, ranging from 2 in Arkansas to 9 in New York (Table 4). Among these genes, several were associated with biological functions of interest. The gene CPA3, which overlapped with outliers in Arkansas, produces a protein that is released by mast cells and is associated with asthma and allergies (Rebhan et al. 1998). TENM1 (MO, NY) is associated with neural development and stress‐induced behaviour while HTR2A (NY) is a serotonin receptor associated with major depressive disorder, obsessive‐compulsive disorder, and schizophrenia (Rebhan et al. 1998). Finally, DZIP3 (MO) is associated with antigen recognition in the innate immune system and SIK2 (NY) relates to energy metabolism and immune reactivity (Rebhan et al. 1998). GO term analyses indicated there was functional over‐representation among the genes from many analyses (Table S7). Several terms related to the adaptive immune system were overrepresented in Arkansas and Missouri, as were multiple terms associated with nervous system activity in Arkansas and New York (Table 5).

**TABLE 4:** Genes that contained outlier loci in every threshold‐based analysis of *F*_st_ and SNP allele frequency in each state.

State	GeneID	Gene	Function
AR	ENSMLUG00000010364	AFF2	RNA‐binding, splicing, fragile X syndrome, intellectual disorder
AR	ENSMLUG00000007858	CPA3	Theopental allergy, asthma, metalloprotease, mast cells, protein degradation
MO	ENSMLUG00000014882	TENM1	Expressed in neurons, neural development, autism, regulates stress induced behaviour
MO	ENSMLUG00000013812	FAM117B	Lateral sclerosis, Mast Syndrome
MO	ENSMLUG00000017795	MDH2	Glycolysis, citric acid cycle
MO	ENSMLUG00000003356	DZIP3	Class I MHC antigen processing and presentation, Innate Immune system
NJ	ENSMLUG00000017713	AKAP9	Microtubules, cell cycle, cell movement
NJ	ENSMLUG00000013582	Unknown	Unknown
NJ	ENSMLUG00000006048	SHROOM2	Cell morphology, Actin binding, highly expressed in the retina
NY	ENSMLUG00000009331	DMXL1	Regulatory function, expressed in many tissues particularly the eye, autism
NY	ENSMLUG00000014882	TENM1	Expressed in neurons, neural development, autism, regulates stress induced behaviour
NY	ENSMLUG00000007661	HTR2A	Serotonin receptor, major depressive disorder, obsessive compulsive disorder, schizophrenia
NY	ENSMLUG00000006158	CTPS2	Pyrimidine metabolism, nucleotide synthesis
NY	ENSMLUG00000009541	EXOC6	Vesicle‐mediated transport, organelle biogenesis and maintenance
NY	ENSMLUG00000004162	USP32	Positive regulation of TORC1 signalling, calcium ion binding, protein processing
NY	ENSMLUG00000010657	SIK2	Glucose/energy metabolism, fatty acid oxidation, autophagy, immune system
NY	ENSMLUG00000002665	PTEN	Cancer, AKT Pik signalling, mTOR pathway, prolactin signalling, gene expression, tumour suppressor
NY	ENSMLUG00000017527	FAM120A	VEGFA‐VEGFR2 signalling, autism, cancer, oxidative stress survival response

*Note:* Function is a truncated summary of the reported function for the respective gene in *GeneCards* (Rebhan et al. 1998).

**TABLE 5:** A subset of the significantly enriched GO terms from each state.

State	Analysis	Term	FDR	Size	Query	Intersection
AR	D 50 k	Immunoglobulin mediated immune response	0.0019	290	14	5
AR	D 50 k	B cell mediated immunity	0.0019	291	14	5
AR	D 50 k	Adaptive immune response	0.0285	506	14	5
AR	D 50 k	Impaired glucose tolerance	0.0093	41	6	3
AR	D 50 k	Insulin‐resistant diabetes mellitus	0.0100	42	6	3
AR	D 50 k	Overweight	0.0241	56	6	3
AR	D 50 k	Neonatal insulin‐dependent diabetes mellitus	0.0018	24	6	3
AR	D 50 k	Hypoinsulinemia	0.0020	25	6	3
AR	SNP	Neuron projection	< 0.0001	705	223	27
AR	SNP	Axon	0.0001	291	223	16
MO	*F*_st_ 50 k	Antigen processing and presentation, exogenous lipid antigen via MHC class Ib	0.0047	18	31	3
MO	*F*_st_ 50 K	Antigen processing and presentation of exogenous antigen	0.0312	33	31	3
MO	SNP	Nervous system development	0.0207	1375	308	45
MO	SNP	Neuron to neuron synapse	0.0030	159	323	12
MO	SNP	Neuron projection	0.0040	705	323	28
MO	SNP	Postsynaptic density	0.0045	139	323	11
NY	SNP	Nervous system development	< 0.0001	1375	738	105
NY	SNP	Neurogenesis	< 0.0001	1000	738	79
NY	SNP	Postsynaptic density	0.0132	139	786	17

*Note:* The state each result came from is designated by the two‐letter state code. The analysis in which the GO term was enriched is designated as *F*_st_ for the genomic regions with outlier values of *F*_st_, D for those with above‐threshold values of Tajima's D, and SNP for loci with outlier changes in allele frequency. The 10 k or 50 k designates whether the analysis included 10,000 bp windows with a 2000 bp step or 50,000 bp windows with a 10,000 bp step. The FDR is the Benjamini‐Hochberg adjusted *p*‐value. Size refers to the number of genes containing that GO term in the genome, Query refers to the number of genes input into g:Profiler, and Intersection refers to the number of genes in the query list that possessed the GO term.

Scans for parallel selection uncovered no outlier genomic regions or SNPs that were shared among all states. We also found no genomic regions in the 99th percentile of *F*_st_ that were shared among all states at either window size. There were 10–15 SNPs in the 99th percentile of change in every state among quality iterations but this was not more than would be expected by chance based on the hypergeometric test (Table S8).

Effective population size as calculated by the Jorde‐Ryman method was low for every state (Table S9). Assuming a generation time of 6 years, N_e_ ranged from 79 in the most lenient iteration of New Jersey to 203 in the most stringent iteration of Arkansas. Across quality iterations, the calculated values of N_e_ remained relatively stable, with an average standard deviation of 14.1. Decreasing the generation time to 3 years resulted in an approximate doubling of estimated N_e_, the lowest again in the most lenient iteration of New Jersey (158) and the highest in the most stringent iteration of Arkansas (406). Regardless of the value of N_e_ used or the quality iteration of the SNP call, the cmh test produced 0 loci with an FDR < 0.2 when including all states.

**FIGURE 4:**
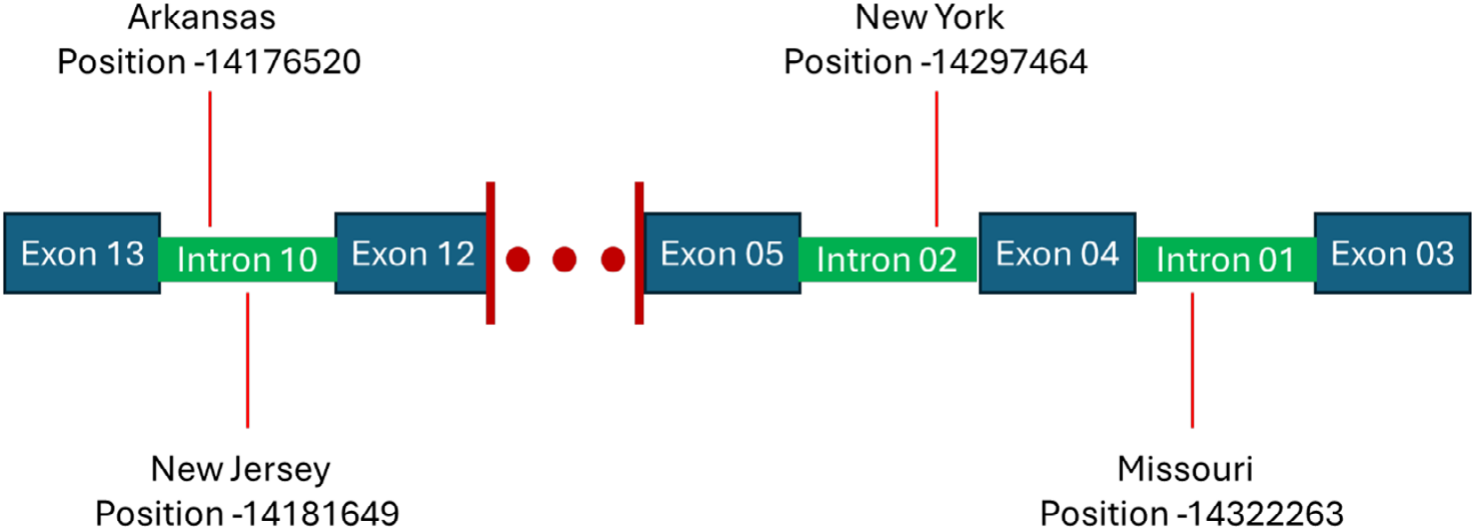
Position of SNPs from each state that had allele frequency changes > 5 standard deviations above the mean within the gene GRIA3. Exons and introns are numbered as they are in the MyoLuc2.0 genome in NCBI. The red lines point to the approximate position of the SNPs, denoting the state in which each SNP was an outlier and the base pair position.

Despite the lack of evidence of parallel selection on the same genomic loci, we found that 3 genes contained SNPs with outlier changes in allele frequency in every state (Figure 4). Two of those genes (ENSMLUG00000010646 and ENSMLUG00000000264) have unknown functions with possible links to microRNAs. The third, GRIA3, is a glutamate receptor considered the primary excitatory neurotransmitter receptor in the mammalian brain (Rebhan et al. 1998). Such receptors contribute to a wide variety of typical neurological functions including the process of long‐term potentiation during memory formation (Rebhan et al. 1998). GRIA3 has been implicated in multiple intellectual developmental disorders and depression (Rebhan et al. 1998). In addition, 11 GO terms were overrepresented among genes with overlapping outlier SNPs in every state (Table S10). However, the functions of these GO terms were vague, including “transmembrane transporter activity” and “cytoplasm”, making them difficult to link to higher‐order physiology.

The cmh test of the two Indiana bat recovery units, Northeast and Ozarks, revealed some significant loci in each state that depended on quality iteration, N_e_, and generation time (Table S11). In the most lenient iteration of the Northeast analysis (75% missingness threshold, lenient SNP quality filter), 8–14 SNPs had an FDR < 0.2 in every iteration of N_e_ and generation time, with 8 loci meeting the threshold in every version. In the strictest iteration of the Northeast analysis, no loci possessed an FDR < 0.2 until N_e_ was at least 20% of census population size when generation time was at 6 years and not until N_e_ was fixed at 1000 for both states when generation time was at 3 years. After those respective N_e_ levels, 3 loci were significant in each iteration (Table 6). For the Ozarks region, no analysis produced loci with FDR < 0.2 until N_e_ was at least 5% of census, with that number climbing to 20% of census in the more lenient iteration with a generation time of 6 years. In the Ozarks, the number of significant loci ranged from 1 to 4 with 1 being the highest in the more stringent version of the analysis.

**TABLE 6:** The results from the CMH test for the recovery unit analysis.

Recovery Unit	Missingness	Stringency	Generations	*N*_e_	Scaffold	Position	FDR
OZ	75	Lenient	6,3	3,2	GL429769	4,377,138	0.194
OZ	75	Lenient	6,3	3,2	GL429769	27,291,180	0.194
OZ	75	Lenient	6,3	3,2	GL430014	854,066	0.194
OZ	75	Lenient	6,3	3,2	GL432451	5723	0.194
OZ	25	Stringent	6,3	5,5	AAPE02068857	5455	0.155
NE	75	Lenient	6,3	6,6	GL429767	56,573,935	0.005
NE	75	Lenient	6,3	6,6	GL429776	14,739,472	0.142
NE	75	Lenient	6,3	6,6	GL429826	5,844,730	0.067
NE	75	Lenient	6,3	6,6	GL429988	2,063,743	0.067
NE	75	Lenient	6,3	6,6	GL430038	1,595,293	0.045
NE	75	Lenient	6,3	6,6	GL430062	180,442	0.002
NE	75	Lenient	6,3	6,6	GL430161	562,703	0.009
NE	75	Lenient	6,3	6,6	GL430912	4056	0.049
NE	25	Stringent	6,3	3,2	GL430018	1,370,457	0.200
NE	25	Stringent	6,3	3,2	GL430062	180,442	0.200
NE	25	Stringent	6,3	3,2	GL430141	78,814	0.200
NE	25	Stringent	6,3	3,2	GL430141	679,952	0.200

*Note:* The first column shows whether the analysis included samples from the Northeast (NE) or Ozarks (OZ) recovery unit. The columns labelled missingness and stringency refer to the quality iteration of the analysis. The generations column shows which value of generation time (6 years or 3 years) resulted in the significant SNP, where 6,3 implies the locus was significant in analyses assuming both a 6 year generation time and those assuming a 3 year generation time. The N_e_ column shows how many of the 6 N_e_s we assumed for each value of generation resulted in the significant SNP. So, 3,2 in that column means the SNP was significant for 3 values of N_e_ when assuming a 6 year generation time and 2 values of N_e_ when assuming a 3 year generation time. Scaffold and position refer to the genomic coordinates of the significant SNP. FDR is the Benjamini‐Hochberg adjusted *p*‐value.

Among states, we found low similarity in the outlier genomic regions and SNPs. Every pairwise comparison demonstrated average Jaccard similarity values lower than 0.01 except for the relationship between Arkansas and New York. The highest similarity was between Arkansas and New York; although the magnitude of this relationship was still modest, at 0.014 (Figure S4).

## 4 Discussion

In this study, we found evidence of some population structure in Indiana bats, but no evidence of a strong population bottleneck either before or after WNS emergence. Our results imply high genetic connectivity across the Indiana bat range, except for one hibernaculum in northern New York. We also see very little evidence of disease‐induced parallel selection, with the exception being a handful of loci in subsets of states, at different loci within three genes, and 11 shared functional relationships among genes containing outlier loci. However, we did observe outlier loci within genes that have functions pertaining to the immune system and energy metabolism as well as functional over‐representation of immune activity, energy metabolism, and nervous system development within individual states. Although it is difficult to directly attribute non‐parallel allele frequency changes to WNS‐induced selection, some of these signals do have functional significance to WNS.

Previous population genetics studies of Indiana bats report little substructure and high gene flow throughout the range (Nagel et al. 2023; Vonhof et al. 2016). Our results also support high gene flow across study states given close clustering of most states in our population analyses and the rapid rate of linkage disequilibrium decay (Loh et al. 2013). However, mitochondrial analysis suggests the existence of five matrilineal clusters across the United States, with more pronounced differences in the northeast (Vonhof et al. 2016). We found that bats from northern New York were the most differentiated from other populations, including geographically close hibernacula in southern New York and New Jersey. The geographic “cut‐off” point we detected mirrors findings from a previous study indicating that northern New York hibernation sites demonstrate genetic separation from southern New York and all sites further south (Vonhof et al. 2016). This separation may be due to non‐overlapping migration patterns in the region resulting in reduced gene flow between northern New York hibernacula and those further south, a conclusion supported by studies of Indiana bat dispersal after spring emergence. Data confirm that ~75% of bats from Barton Hill Mine in northern New York moved only to the Lake Champlain valley at the border of New York and Vermont (Carl Herzog, New York Department of Environmental Conservation, personal communication). We did not see evidence of population structure changing to any significant degree in response to WNS, implying mating and migration behavior were maintained despite the reduction in population size. Further study of Indiana bat population genetic structure, particularly in the northeastern United States, could help identify conservation units that display unique patterns of disease spread and inform effective management of this endangered species.

The negative genome‐wide values of Tajima's D we observed in every state and the enrichment of rare alleles in the allele frequency spectra suggest there was no strong bottleneck impacting Indiana bat demographics in either time period (Charlesworth 2006; Eckshtain‐Levi et al. 2018; Fijarczyk and Babik 2015; Sodeland et al. 2022; Tajima 1989). It may even indicate recent population expansion, possibly due to the noted census increases in the early 2000s. However, we interpret the excess of rare alleles with caution because we are using low‐coverage whole‐genome data, which can inflate the number of sequencing errors among rare alleles. We also did not observe a large change in the site frequency spectra or genome‐wide Tajima's D between pre‐ and post‐WNS sample groups, at least among all samples or most states. The only exceptions to the lack of change between time periods were in Missouri and New Jersey, where post‐WNS samples had higher values of Tajima's D and a relative dearth of rare alleles. In both states, Tajima's D was still negative, but the increase may indicate population reduction after WNS emergence in these areas. The total sample group and New York also demonstrated reductions in Tajima's D after WNS emergence, but these changes were relatively slight and did not show up in the site frequency spectra. However, Tajima's D actually decreased after WNS emergence in Arkansas, supporting population expansion or recovery in this location. These results indicate potential site‐specific demographic impacts of WNS on Indiana bats, which would likely be due to the noted variability in disease impacts among hibernacula in this species (Hoyt et al. 2021; Thogmartin et al. 2012).

In addition to the lack of a disease‐induced bottleneck, we found very little evidence of parallel selection occurring in Indiana bats, at least across all states in the study. The only exceptions to this finding are in three genes that contain outlier loci in every state and 11 GO terms that were enriched among genes containing outlier loci in every state. The GO terms, however, were largely uninformative, as they included functions like transporter activity and membrane that are difficult to link to specific physiological processes. Similarly, two of the three genes containing outlier loci in every state were unnamed and had unknown function. The third gene, GRIA3, is a glutamate receptor that is thought to be the predominant excitatory neurotransmitter receptor in the mammalian brain (Rebhan et al. 1998). It may be that the different loci under selection among states in these genes have redundant functional impacts that lead to similar phenotypic outcomes. Given this finding, it is possible other examples of variable changes in genetic coordinates leading to similar phenotypic consequences exist in Indiana bats that we were unable to detect. We cannot rule out a highly polygenic evolutionary response to WNS in Indiana bats.

Given the short time‐frame over which we attempted to detect parallel selection in this study, it is possible selection is occurring but has not yet produced a detectable signal. The post‐WNS samples were all collected within two generations of WNS emergence. Later sampling efforts may be better suited to detect a signal of selection given Indiana bats do not appear to have undergone a strong bottleneck. However, similar studies of the closely related little brown bat ( *Myotis lucifugus* ) sampled along a similar time frame were able to detect selection (Auteri and Knowles 2020; Donaldson et al. 2017; Gignoux‐Wolfsohn et al. 2021).

Contrasting evolutionary responses in these two closely related bat species may result from differential mortality rates and/or the diversity of initial standing genetic variation. Little brown bats experienced more drastic disease‐induced declines than Indiana bats (Cheng et al. 2021; Hoyt et al. 2021), which likely produced a more intense selection pressure and higher likelihood of detectable rapid adaptation. Pre‐WNS Indiana bat populations were also smaller than those of little brown bats (Hoyt et al. 2021), likely leading to lower overall genetic diversity and thus a lower chance of individuals possessing beneficial alleles. In other words, Indiana bats may have had lower evolutionary potential than little brown bats.

Our findings also suggest that parallel selection may be occurring in subsets of states, supporting the hypothesis that fitness benefits of alleles vary based on environmental factors or that the magnitude of selective pressure due to WNS varies by region. We found several SNPs that significantly differed between pre‐ and post‐WNS samples in parallel within the Northeast and Ozarks recovery units. There were more significant loci in the Northeast supporting past evidence that WNS impact was greater in the Northeast (Thogmartin et al. 2012, 2013), possibly leading to higher selective pressure. However, the difference in the number of loci under selection between the Northeast and Ozarks in the current study was small and depended on N_e_, which should be considered in the interpretation of these results. Surprisingly, we saw the greatest similarity among outlier loci between New York and Arkansas. These locations are the furthest apart geographically, and our population genetic analysis suggests they are not more genetically similar than other states. This suggests that there may be similar impacts of WNS or some unmeasured environmental variable to which Indiana bats in Arkansas and New York are responding. However, the suite of outlier loci in each of these two states was still quite dissimilar (Jaccard similarity < 0.2), reinforcing the fact that there was very little overlap in outlier loci among states.

In every state we analysed, there were numerous genomic coordinates presenting outlier changes in allele frequency possibly caused by selection. It is difficult to be certain of the cause or even if the changes are due to selection at all within any given state without the support of parallel changes in other sites. Genetic drift can result in large changes in allele frequency that can obscure signals of selection and create false positives. When we attempted to identify outlier genomic regions between pre‐ and post‐WNS samples using a null model of *F*_st_, we found no outlier regions. This negative finding and the lack of parallel changes across states support the idea that many of the outlier loci we detected may be false positives attributable to genetic drift. Further, even if these loci are under selection, it is not guaranteed that the selection is due to WNS. However, strong environmental selection pressure can result in heterogeneous evolution over a landscape, even in closely associated populations with high gene flow (Carvalho et al. 2022; Choubisa and Choubisa 2021; Lee and Mitchell‐Olds 2011; Liao et al. 2021; McEvoy et al. 2009). In addition, population trends in response to WNS were widely variable among hibernation sites (Thogmartin et al. 2012, 2013), potentially leading to variable selection regimes across sites. Past work identified a positive relationship between Indiana bat mortality rates and hibernaculum humidity, suggesting more favorable growth conditions for *P. destructans* lead to lower host survival (Langwig et al. 2012). It has also been hypothesised that hibernaculum temperature and bat microclimate selection within hibernacula could be partially responsible for differential mortality rates (Hoyt et al. 2021; Langwig et al. 2012). Therefore, hibernaculum factors have the potential to influence the degree of WNS‐induced selection pressure. Supporting a variable selection regime, our analysis of genome‐wide Tajima's D and site frequency spectra suggests variable demographic impacts of WNS among states. Although we cannot be certain of the degree to which the per‐state outlier loci are under disease‐induced selection, we still highlight those results because some of the genes containing outlier loci had functional annotations with potential relevance to WNS pathophysiology.

Genes and GO terms with relationships to the immune system seem most likely to be directly related to WNS. In Arkansas, the gene CPA3 and in Missouri, the gene DZIP3, contained outlier genomic loci in every threshold‐based analysis of *F*_st_ and SNPs. CPA3 is a metalloprotease stored in mast cells and is associated with inflammatory responses underlying allergic and asthmatic reactions in humans when released (Pejler 2019; Siddhuraj et al. 2022). DZIP3 codes for a protein that epigenetically modifies histone H2A in a way that represses the transcription of a subgroup of chemokines; removal of this modification is necessary for the intracellular propagation of pro‐inflammatory signals (Zhang and Cao 2019; Zhou et al. 2008). Four terms related to the immunoglobulin‐mediated immune response and five terms related to adaptive immunity were significantly overrepresented among genes containing outlier loci in Arkansas. In Missouri, four terms related to antigen processing and presentation were enriched among genes containing outlier loci. Past work in little brown bats has shown that the transcriptomic response to WNS involves cytokine release through antigen recognition receptors, systemic inflammation, and the activation of a partial adaptive immune response during torpor arousal (Davy et al. 2020; Field et al. 2018; Kwait et al. 2025; Lilley et al. 2019; Meteyer et al. 2012). In addition, several intergenic loci under selection in little brown bats regulate the expression of genes associated with the immune and inflammatory response (Gignoux‐Wolfsohn et al. 2021; Kwait et al. 2024), and multiple immune genes show signs of selection due to WNS in little brown bats (Donaldson et al. 2017). Our results suggest Indiana bats are potentially undergoing similar forms of adaptation, where alteration of immune activity resulting from selection within some genes may affect the ability of Indiana bats to clear infection (i.e., resistance) or their ability to survive despite continued infection (i.e., tolerance). Past studies of immune responses to WNS support both resistance and tolerance as strategies employed by species that persist despite the presence of *P. destructans* (Cheng et al. 2024; Davy et al. 2020; Kwait et al. 2024; Lilley et al. 2019; Whiting‐Fawcett et al. 2024).

Potentially relating to a tolerance strategy of disease survival, one gene in New York and multiple GO terms in Arkansas were part of processes related to food intake and energy cycling. In New York, the gene SIK2 contained outlier loci in every threshold‐based analysis of *F*_st_ and SNPs. SIK2 is a salt‐inducible kinase that is highly expressed within adipocytes and regulates glucose uptake into the cell (Henriksson et al. 2015). In Arkansas, 16 GO terms related to diabetes, glucose cycling, insulin cycling or weight gain were enriched among genes containing outlier loci. In humans insulin resistance is a common consequence of obesity and itself promotes obesity and diabetes (Kahn and Flier 2000). Reversible insulin resistance mirroring diabetes is part of the physiological mechanism responsible for fall hyperphagia and fattening in pre‐hibernating mammals (S. L. Martin 2008; Wu et al. 2013). Insulin and insulin resistance also vary over the course of the hibernation season, implying insulin plays a role in energy cycling during torpor (Bauman 1990; Boswell et al. 1994; Rigano et al. 2017). Depletion of fat reserves due to an increased frequency of arousal from torpor is thought to be among the primary causes of mortality in bats with WNS (Cheng et al. 2021; Hoyt et al. 2021; Reeder et al. 2012). If changes in these genes impact the amount of fat Indiana bats accrue during pre‐hibernation hyperphagia or reduce energy use during the hibernation season, they could help buffer individuals against energy loss from WNS. In little brown bats, post‐WNS populations have higher fat mass (Cheng et al. 2019) and multiple loci found to be under selection due to WNS are in or near genes related to glucose cycling, insulin, or diabetes (Auteri and Knowles 2020; Gignoux‐Wolfsohn et al. 2021; Lilley et al. 2020). In addition, some of the loci under selection in little brown bats impact the expression of genes related to insulin, glucose, and energy cycling (Kwait et al. 2024). The results of these past studies suggest the accrual of fat mass and energy cycling during hibernation are under selection due to WNS in little brown bats. It is possible Indiana bats are responding similarly in parts of their range.

Finally, there were genes and GO terms with relationships to nervous system development, neurotransmitter receptors, and neurological disorders in every state but New Jersey. One of the genes potentially under parallel selection, GRIA3, is one of the primary neurotransmitter receptors in the mammalian brain (Rebhan et al. 1998). One gene in Arkansas, one in Missouri, and four in New York contained outlier loci in every threshold‐based analysis of *F*_st_ or SNPs and have some relation to autism, intellectual disorders, or neural development (Rebhan et al. 1998). One of those genes from New York, HTR2A, is a serotonin receptor that has been implicated in multiple psychological disorders including major depressive disorder and obsessive‐compulsive disorder (Kao et al. 2020; Zamanian‐Azodi et al. 2021; Zhao et al. 2014). Eight over‐represented GO terms in Arkansas, ten in Missouri, and 28 in New York were associated with neurological development or activity. It is possible selection in these genes is impacting hibernation physiology in Indiana bats. Neurons reduce in complexity during torpor, likely to preserve energy, and then regrow upon arousal (Mohr et al. 2020). Polymorphism in molecular neural pathways may impact the energetic requirements or magnitude of neuronal contraction in Indiana bats during hibernation. Given the primary mechanism leading to WNS mortality is an increase in torpor arousal frequency (Reeder et al. 2012), it is possible that selection within these pathways impacts the odds of survival for infected individuals. However, a more direct link between these processes is necessary to test this hypothesis.

From a management perspective, it appears that Indiana bats are not likely experiencing range‐wide rapid adaptation. Similarly, targeted translocation of individuals with alleles thought to be beneficial is unlikely to be widely successful. Our results suggest limited evidence of parallel adaptation but potential recovery unit‐specific selection, with the Northeast possessing more SNPs under selection. We also uncovered evidence of state‐specific demographic impacts of WNS in the form of varying directional shifts in genome‐wide Tajima's D and the proportion of rare alleles. We advise that management of Indiana bats should include colony‐specifc considerations given variation in both population growth trajectories and signals of selection. We should also not assume that declines in a colony are associated with WNS. In some locations, it is possible that differential survival and selection pressure are completely unrelated to WNS. Although there was recovery of Indiana bat population growth rates in many locations in the early 2000s, some hibernacula were still experiencing declines prior to WNS emergence (Thogmartin et al. 2012; USFW 2007, 2009). We also did not observe evidence of a WNS associated population bottleneck in most states. Therefore, some declines may be explained by other factors such as deforestation, pesticide use, and hibernaculum disturbance.

Future studies of selection in Indiana bats could further disentangle some of the patterns we have uncovered here. Including more states could help determine if any broad environmental or demographic patterns relate to the likelihood of parallel selection occurring. Further, investigating relationships among hibernaculum microclimate, bat genotype, and WNS survival may elucidate confounding factors that influence selection. Connecting some of the genomic coordinates we identified as under selection to phenotypic function would both validate the relevance of these loci to WNS and provide information regarding the effect of polymorphism in these loci (Cullingham et al. 2020; Kwait et al. 2024). In addition, the pathophysiology of WNS has not been thoroughly investigated in Indiana bats, and past studies demonstrate that species' physiological reactions to infection are variable (Davy et al. 2020; Lilley et al. 2019). As such, studies of hibernation pattern and pathophysiology in response to infection would be valuable. Finally, investigating other mechanisms of host response, such as antibodies for *P. destructans* consistent with an adaptive immune response, may provide additional insight into how to most effectively manage Indiana bats affected by WNS.

## 5 Conclusion

In this study of Indiana bats, we assessed population structure, searched for population bottlenecks, and for signatures of selection associated with WNS emergence. Congruent with past work, we found evidence of population substructure with one northern New York hibernation site separating from the rest of the species' range. Genome‐wide Tajima's D and allele frequency patterns indicate no evidence of a population bottleneck prior to or after WNS across all samples and in most states, suggesting WNS did not strongly impact Indiana bat demographics. However, changes in Tajima's D and the site frequency spectra after WNS varied by state, suggesting potential site‐specific demographic impacts of WNS. We found little evidence of range‐wide parallel selection; however, parallel selection may be occurring in subsets of states and on different loci within three genes. Lastly, we found that outlier loci in individual states were within genes reporting functions associated with immune responses, energy cycling, and neural development. Although it is difficult to directly link these outlier loci to WNS without parallel changes among sites, it is possible they relate to WNS pathophysiology and have undergone selection only in certain regions due to the variability in disease impacts among sites.

## Author Contributions

All authors contributed to the conception and design of this study. Sample collection was led by Maarten Vonhof, Brooke Maslo, Brooke Hines, and Evan A. Eskew. Laboratory work was performed by Robert Kwait and advised by Sarah Gignoux‐Wolfsohn, Evan A. Eskew, and Malin L. Pinsky. Data analysis was performed by Robert Kwait and advised by Malin L. Pinsky, Evan A. Eskew, Sarah Gignoux‐Wolfsohn, Maarten Vonhof, and Brooke Maslo. The first draft of this manuscript was written by Robert Kwait and the editing effort was led by Brooke Maslo. All authors contributed to writing edits in subsequent versions. All authors read and approved the final manuscript.

## Conflicts of Interest

The authors declare no conflicts of interest.

## Supporting information


**Data S1:** mec70172‐sup‐0001‐Supinfo.zip.

## Data Availability

All sequences generated as part of this project are publicly available through NCBI (BioProject: PRJNA1354827). All code for data analysis is available on Github (BKrek89/M.‐sodalis‐LcWGS).
