# Supplementary material for: Low‐Coverage Whole‐Genome Analysis of Population Structure, Bottlenecks, and Selection in Indiana Bats Before and After White‐Nose Syndrome
Source: Mol Ecol. 2025 Nov 10;34(24):e70172. doi: 10.1111/mec.70172 (PMC12717984; doi:10.1111/mec.70172)
Supplement: Supplementary file 1 — Data S1: mec70172‐sup‐0001‐Supinfo.zip. [file MEC-34-e70172-s001.zip › Supplementary Table 1-11 Figure 1-4.docx]

| **State** | **County** | **Hibernaculum** | **Collection Date** | **Sample #** |
| --- | --- | --- | --- | --- |
| Arkansas | Newton | Cave Mountain Cave | 2000 | 3 |
| Arkansas | Newton | Edgeman Cave | 2000 | 5 |
| Arkansas | Stone | Roland Cave | 2000 | 9 |
| Arkansas | Stone | Gustafson Cave | 2000 | 3 |
| Arkansas | Stone | Amphitheater Cave | 2000 | 8 |
| Arkansas | Newton | Edgeman Cave | 2019 | 34 |
| Kentucky | Carter | Carter Co. Cave | 1997 | 36 |
| Kentucky | Madison | Colossal Cave | 2017 | 27 |
| Kentucky | Rockcastle | Salt Peter Cave | 2020 | 17 |
| Missouri | Iron | Pilot Knob Mine | 1998-1999 | 35 |
| Missouri | Iron | Cave Hollow Cave | 1998 | 2 |
| Missouri | Pulaski | Great Spirit Cave | 1998 | 4 |
| Missouri | Shannon | Bat Cave | 1998 | 1 |
| Missouri | Washington | Great Scott Cave | 1998 | 4 |
| Missouri | Washington | Great Scott Cave | 2020 | 30 |
| New Jersey | Morris | Hibernia Mine | 2006 | 10 |
| New Jersey | Morris | Mt. Hope West Mine | 2006 | 18 |
| New Jersey | Morris | Mt. Hope West Mine | 2021 | 29 |
| New York | Essex | Barton Hill Mine | 1999-2009 | 31 |
| New York | Ulster | Walter Williams Preserve Mine | 2008-2009 | 4 |
| New York | Essex | Barton Hill Mine | 2020 | 30 |

**Supplementary Table 1**. The samples collected in our study by hibernaculum, including the county and state of collection. Grayed rows are pre-WNS samples and unhighlighted rows are post-WNS samples.

| **State** | **Time Period** | **Sample #** | **Average Coverage** | **% genome** | **% uncalled** | **% missing** |
| --- | --- | --- | --- | --- | --- | --- |
| AR | Post-WNS | 34 | 78.6 | 74.9 | 0.082 | 16.9 |
| AR | Pre-WNS | 26 | 36.1 | 64.3 | 0.088 | 28.9 |
| KY | Post-WNS | 39 | 65.5 | 66.3 | 0.211 | 26.5 |
| KY | Pre-WNS | 30 | 17.3 | 30.6 | 0.208 | 46.5 |
| MO | Post-WNS | 30 | 44.9 | 67.8 | 0.112 | 24.9 |
| MO | Pre-WNS | 46 | 77.5 | 67.1 | 0.119 | 25.7 |
| NJ | Post-WNS | 27 | 34.1 | 64.9 | 0.084 | 27.2 |
| NJ | Pre-WNS | 32 | 49.8 | 68.7 | 0.083 | 23.8 |
| NY | Post-WNS | 30 | 64.9 | 75.4 | 0.072 | 16.4 |
| NY | Pre-WNS | 33 | 49.9 | 71.3 | 0.073 | 20.8 |

**Supplementary Table 2**. Quality statistics for each sample group after sequencing. Sample groups are denoted by the two-letter state code. Average coverage refers to the low coverage whole-genome convention of coverage or the average x coverage in each sample group multiplied by the number of samples in that group. % genome shows the average percentage of the genome containing reads for each sample. % uncalled refers to the average number of bases called as N, or those not assigned to a nucleotide, during sequencing. % missing depicts the average percent of loci that were not called per sample.

| **Filtering step** | **Results** | **Values** |
| --- | --- | --- |
| Sample Selection and Sequencing | Number of Samples Collected | 340 |
|  | Number of Samples Sequenced | 327 |
| Sequence QC | Number of Samples Successfully Sequenced | 243 |
|  | Raw Read Count | 15434053712 |
|  | Reads Retained after Quality Filtering | 12652863105 |
| Mapped Read Filtering | Number of Reads that Mapped | 10491066822 |
|  | Number of Reads Remaining after Filtering Improperly Paired | 9060026417 |
|  | Number of Reads Remaining after Filtering for PCR Duplicates | 8733652682 |

**Supplementary Table 3**. Sample number and read counts after key filtering steps prior to variant calling. Values show the count of either samples or reads after the filtering step listed under results. The number of samples successfully sequenced depicts the number of samples with acceptable quality levels, excluding all samples from Kentucky. All read count data excludes samples sourced from Kentucky.

| **Variable** | **Filter** | **Remaining** |
| --- | --- | --- |
| SNP | Initial quality filtering | 6345895 |
|  | Allele balance | 5245285 |
| Sample | No missing threshold | 243 |
|  | Missing threshold 75% and PCA outliers | 220 |
|  | Missing threshold 25% and PCA outliers | 154 |

**Supplementary Table 4**. SNP and sample number after key filtering steps.

|  |  |  | **Samples** | | **SNPs Called** | |
| --- | --- | --- | --- | --- | --- | --- |
| **State** | **Missingness** | **Stringency** | Pre-WNS | Post-WNS | Pre-WNS | Post-WNS |
| AR | 75 | Lenient | 24 | 32 | 5245282 | 5245045 |
| MO | 75 | Lenient | 39 | 20 | 5245003 | 5245283 |
| NJ | 75 | Lenient | 24 | 25 | 5245283 | 5245283 |
| NY | 75 | Lenient | 29 | 27 | 5245279 | 5245276 |
| AR | 75 | Stringent | 24 | 32 | 5132849 | 5235920 |
| MO | 75 | Stringent | 39 | 20 | 5239217 | 4998117 |
| NJ | 75 | Stringent | 24 | 25 | 5208669 | 5098538 |
| NY | 75 | Stringent | 29 | 27 | 5240987 | 5242011 |
| AR | 25 | Lenient | 8 | 23 | 5245083 | 5244078 |
| MO | 25 | Lenient | 26 | 14 | 5244580 | 5245282 |
| NJ | 25 | Lenient | 21 | 13 | 5245282 | 5245198 |
| NY | 25 | Lenient | 26 | 23 | 5245200 | 5244915 |
| AR | 25 | Stringent | 8 | 23 | 5118258 | 5239076 |
| MO | 25 | Stringent | 26 | 14 | 5241061 | 5242564 |
| NJ | 25 | Stringent | 21 | 13 | 5244357 | 5222728 |
| NY | 25 | Stringent | 26 | 23 | 5243742 | 5242813 |

**Supplementary Table 5**. Sample and SNP counts for each state in each quality filter iteration. Missingness refers to the percent missingness threshold for inclusion, with 75 showing samples with more than 75% missingness excluded. Stringency refers to whether the SNP call was performed with a relatively stringent filter (-minInd, -HWE 0.0001) or a more lenient filter.

| **State 1** | **State 2** | **Missingness** | **pre-WNS** | **post-WNS** |
| --- | --- | --- | --- | --- |
| AR | MO | 75 | 0.0100 | 0.0115 |
| AR | NJ | 75 | 0.0139 | 0.0120 |
| AR | NY | 75 | 0.0144 | 0.0127 |
| MO | NJ | 75 | 0.0110 | 0.0149 |
| MO | NY | 75 | 0.0114 | 0.0157 |
| NJ | NY | 75 | 0.0143 | 0.0156 |
| AR | MO | 25 | 0.0226 | 0.0157 |
| AR | NJ | 25 | 0.0264 | 0.0184 |
| AR | NY | 25 | 0.0261 | 0.0149 |
| MO | NJ | 25 | 0.0137 | 0.0228 |
| MO | NY | 25 | 0.0135 | 0.0192 |
| NJ | NY | 25 | 0.0158 | 0.0213 |

**Supplementary Table 6**. Pairwise F_st_ between each state pre- and post-WNS. States are denoted by their two letter code. Missingness reflects the percent missingness threshold (75% or 25%). The pre-WNS and post-WNS columns show the weighted F_st_ between each pair of states pre- and post-WNS.

**Supplementary Table 7**. Full GO term results for all states as output by g:profiler. States are denoted by two letter state codes and the analysis column shows which analysis the GO term was overrepresented in.

| **Missingness** | **Stringency** | **N** | **n** | **k** | **p-value** |
| --- | --- | --- | --- | --- | --- |
| 75 | lenient | 5244913 | 52451, 52452, 52453, 52454 | 15 | 0.9999 |
| 75 | stringent | 4780557 | 51240, 49925, 50681, 52385 | 10 | 0.9999 |
| 25 | lenient | 5243682 | 52439, 52447, 52452, 52449 | 14 | 0.9999 |
| 25 | stringent | 5085736 | 51123, 52387, 52221, 52421 | 10 | 0.9999 |

**Supplementary Table 8**. Parameter values and p-values for every quality iteration of the hypergeometric test. N refers to the total number of SNPs that were present in each state. n refers to the number of SNPs drawn from each state, in this case meaning those in the highest 99^th^ percentile of allele frequency change. k is the number of loci in the 99^th^ percentile shared between all states.

| **State** | **Missingness** | **Stringency** | **Generation Time** | **Ne** | **Lower CI** | **Upper CI** |
| --- | --- | --- | --- | --- | --- | --- |
| AR | 75 | lenient | 6 | 130.0 | 128.7 | 131.4 |
| MO | 75 | lenient | 6 | 146.3 | 144.7 | 147.9 |
| NJ | 75 | lenient | 6 | 79.3 | 78.6 | 80.1 |
| NY | 75 | lenient | 6 | 171.9 | 170.0 | 173.7 |
| AR | 25 | lenient | 6 | 183.9 | 178.6 | 189.4 |
| MO | 25 | lenient | 6 | 177.4 | 174.6 | 180.1 |
| NJ | 25 | lenient | 6 | 79.0 | 78.0 | 80.1 |
| NY | 25 | lenient | 6 | 176.8 | 174.6 | 178.9 |
| AR | 75 | stringent | 6 | 133.7 | 132.2 | 135.2 |
| MO | 75 | stringent | 6 | 156.0 | 154.2 | 157.7 |
| NJ | 75 | stringent | 6 | 82.5 | 81.7 | 83.3 |
| NY | 75 | stringent | 6 | 171.4 | 169.5 | 173.5 |
| AR | 25 | stringent | 6 | 203.3 | 197.3 | 209.9 |
| MO | 25 | stringent | 6 | 177.2 | 174.4 | 180.1 |
| NJ | 25 | stringent | 6 | 80.6 | 79.4 | 81.7 |
| NY | 25 | stringent | 6 | 176.0 | 173.9 | 178.3 |
| AR | 75 | lenient | 3 | 259.6 | 256.7 | 262.2 |
| MO | 75 | lenient | 3 | 292.1 | 289.2 | 295.2 |
| NJ | 75 | lenient | 3 | 158.7 | 157.3 | 160.3 |
| NY | 75 | lenient | 3 | 343.7 | 339.7 | 347.7 |
| AR | 25 | lenient | 3 | 367.4 | 356.8 | 378.1 |
| MO | 25 | lenient | 3 | 354.2 | 348.7 | 359.6 |
| NJ | 25 | lenient | 3 | 178.2 | 156.1 | 160.3 |
| NY | 25 | lenient | 3 | 353.5 | 349.1 | 357.7 |
| AR | 75 | stringent | 3 | 267.0 | 264.4 | 269.8 |
| MO | 75 | stringent | 3 | 311.5 | 308.3 | 315.1 |
| NJ | 75 | stringent | 3 | 164.9 | 163.3 | 166.6 |
| NY | 75 | stringent | 3 | 342.8 | 338.9 | 346.5 |
| AR | 25 | stringent | 3 | 405.8 | 394.4 | 418.4 |
| MO | 25 | stringent | 3 | 354.0 | 348.3 | 359.7 |
| NJ | 25 | stringent | 3 | 161.1 | 159.0 | 163.4 |
| NY | 25 | stringent | 3 | 352.1 | 347.9 | 356.6 |

**Supplementary Table 9**. N_e_ as calculated using the Jorde-Ryman estimator for each state in each quality iteration. N_e_ is the bootstrapped effective population size estimate with 95% confidence intervals.

| **Analysis** | **Term** |
| --- | --- |
| SNP | transmembrane transporter activity |
| SNP | transporter activity |
| SNP | inorganic cation transmembrane transporter activity |
| SNP | localization |
| SNP | membrane |
| SNP | cell periphery |
| SNP | plasma membrane |
| SNP | cell projection |
| SNP | plasma membrane bounded cell projection |
| *SNP | cytoplasm |
| SNP | cytoskeleton |

**Supplementary Table 10**. GO terms that were enriched among genes containing outlier loci in every state. Analysis refers to which analysis each term came from in all states. *In New York, this GO term was overrepresented in genes with both outlier SNPs and F_st_ outlier 10,000 bp windows.

| **Recovery Unit** | **Missingness** | **Stringency** | **Generation** | **Ne** | **SNPs** |
| --- | --- | --- | --- | --- | --- |
| NE | 75 | lenient | 6 | JR | 10 |
| NE | 75 | lenient | 6 | 5% | 8 |
| NE | 75 | lenient | 6 | 10% | 8 |
| NE | 75 | lenient | 6 | 20% | 11 |
| NE | 75 | lenient | 6 | 1000 | 14 |
| NE | 75 | lenient | 6 | 20000 | 14 |
| NE | 75 | lenient | 3 | JR | 10 |
| NE | 75 | lenient | 3 | 5% | 8 |
| NE | 75 | lenient | 3 | 10% | 8 |
| NE | 75 | lenient | 3 | 20% | 8 |
| NE | 75 | lenient | 3 | 1000 | 14 |
| NE | 75 | lenient | 3 | 20000 | 14 |
| NE | 25 | stringent | 6 | JR | 0 |
| NE | 25 | stringent | 6 | 5% | 0 |
| NE | 25 | stringent | 6 | 10% | 0 |
| NE | 25 | stringent | 6 | 20% | 4 |
| NE | 25 | stringent | 6 | 1000 | 4 |
| NE | 25 | stringent | 6 | 20000 | 4 |
| NE | 25 | stringent | 3 | JR | 0 |
| NE | 25 | stringent | 3 | 5% | 0 |
| NE | 25 | stringent | 3 | 10% | 0 |
| NE | 25 | stringent | 3 | 20% | 0 |
| NE | 25 | stringent | 3 | 1000 | 4 |
| NE | 25 | stringent | 3 | 20000 | 4 |
| OZ | 75 | lenient | 6 | JR | 0 |
| OZ | 75 | lenient | 6 | 5% | 0 |
| OZ | 75 | lenient | 6 | 10% | 4 |
| OZ | 75 | lenient | 6 | 20% | 4 |
| OZ | 75 | lenient | 6 | 1000 | 0 |
| OZ | 75 | lenient | 6 | 20000 | 4 |
| OZ | 75 | lenient | 3 | JR | 0 |
| OZ | 75 | lenient | 3 | 5% | 0 |
| OZ | 75 | lenient | 3 | 10% | 0 |
| OZ | 75 | lenient | 3 | 20% | 4 |
| OZ | 75 | lenient | 3 | 1000 | 0 |
| OZ | 75 | lenient | 3 | 20000 | 4 |
| OZ | 25 | stringent | 6 | JR | 0 |
| OZ | 25 | stringent | 6 | 5% | 1 |
| OZ | 25 | stringent | 6 | 10% | 1 |
| OZ | 25 | stringent | 6 | 20% | 1 |
| OZ | 25 | stringent | 6 | 1000 | 1 |
| OZ | 25 | stringent | 6 | 20000 | 1 |
| OZ | 25 | stringent | 3 | JR | 0 |
| OZ | 25 | stringent | 3 | 5% | 1 |
| OZ | 25 | stringent | 3 | 10% | 1 |
| OZ | 25 | stringent | 3 | 20% | 1 |
| OZ | 25 | stringent | 3 | 1000 | 1 |
| OZ | 25 | stringent | 3 | 20000 | 1 |

**Supplementary Table 11**. Results from CMH test of the recovery subunits. The first column shows whether the test was performed on the samples from the Northeast (NE) or Ozarks (OZ) recovery units. Missingness and stringency refer to the quality iteration. Generation shows the assumed generation time for the analysis. Ne shows which value of Ne was used for the analysis, with JR referring to the Jorde-Ryman estimate for the respective generation time. The percentages show the % of census population in each iteration. 1000 and 20000 refer to runs where all locations were assumed to have effective population sizes of 1000 or 20000. Lastly SNPs shows the number of SNPs for each analysis that had an FDR < 0.2.


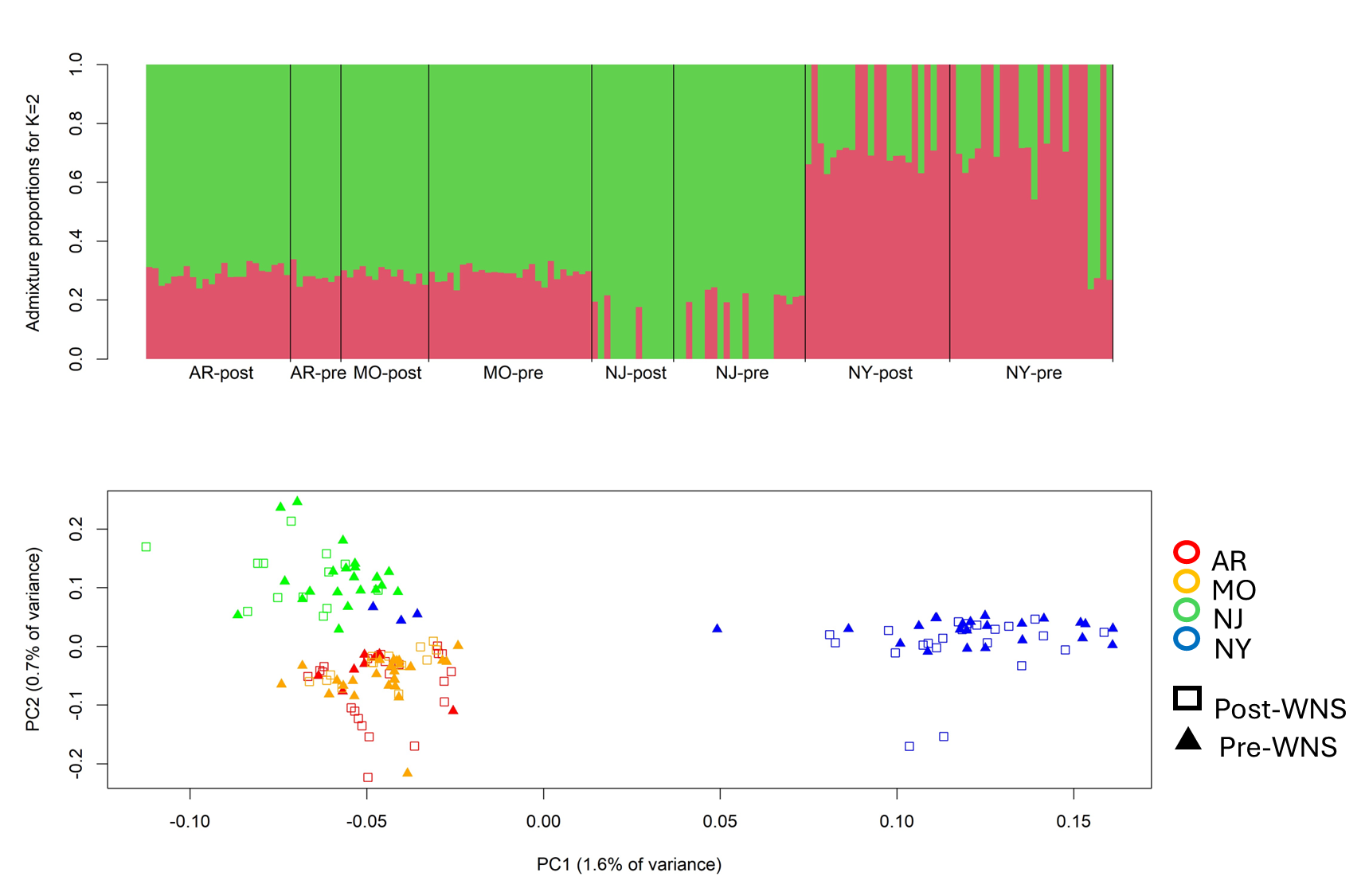


**Supplementary Figure 1**. PCA and admixture plots for analyses employing a missingness threshold of 25%.


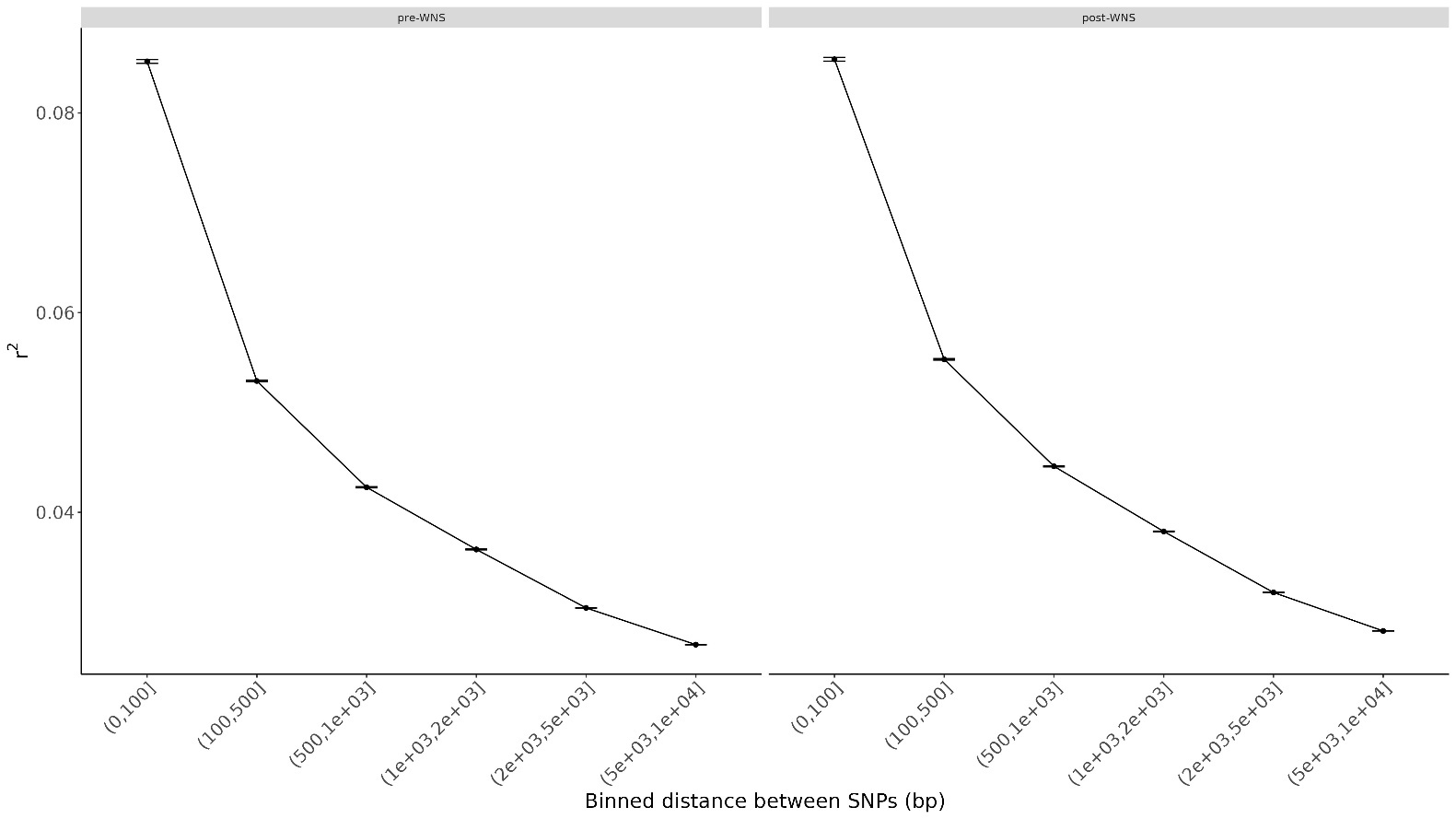


**Supplementary Figure 2**. Linkage disequilibrium decay plot including all samples from both time periods. A) Pre-WNS samples and B) Post-WNS samples. The x-axis is the distance between loci in base pairs binned between threshold values. The y-axis is the average r^2^ value for the linkage relationships between SNPs for each bin with 95% confidence intervals.


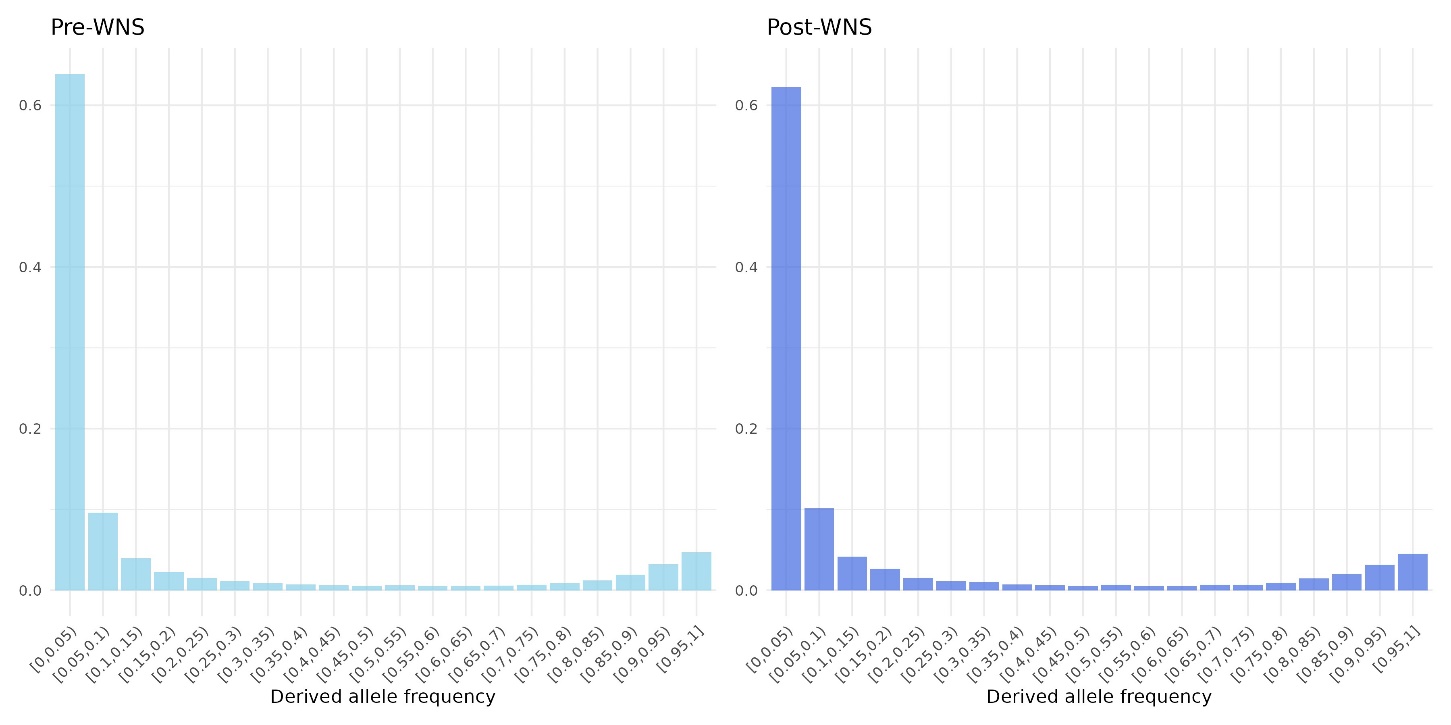


**All**


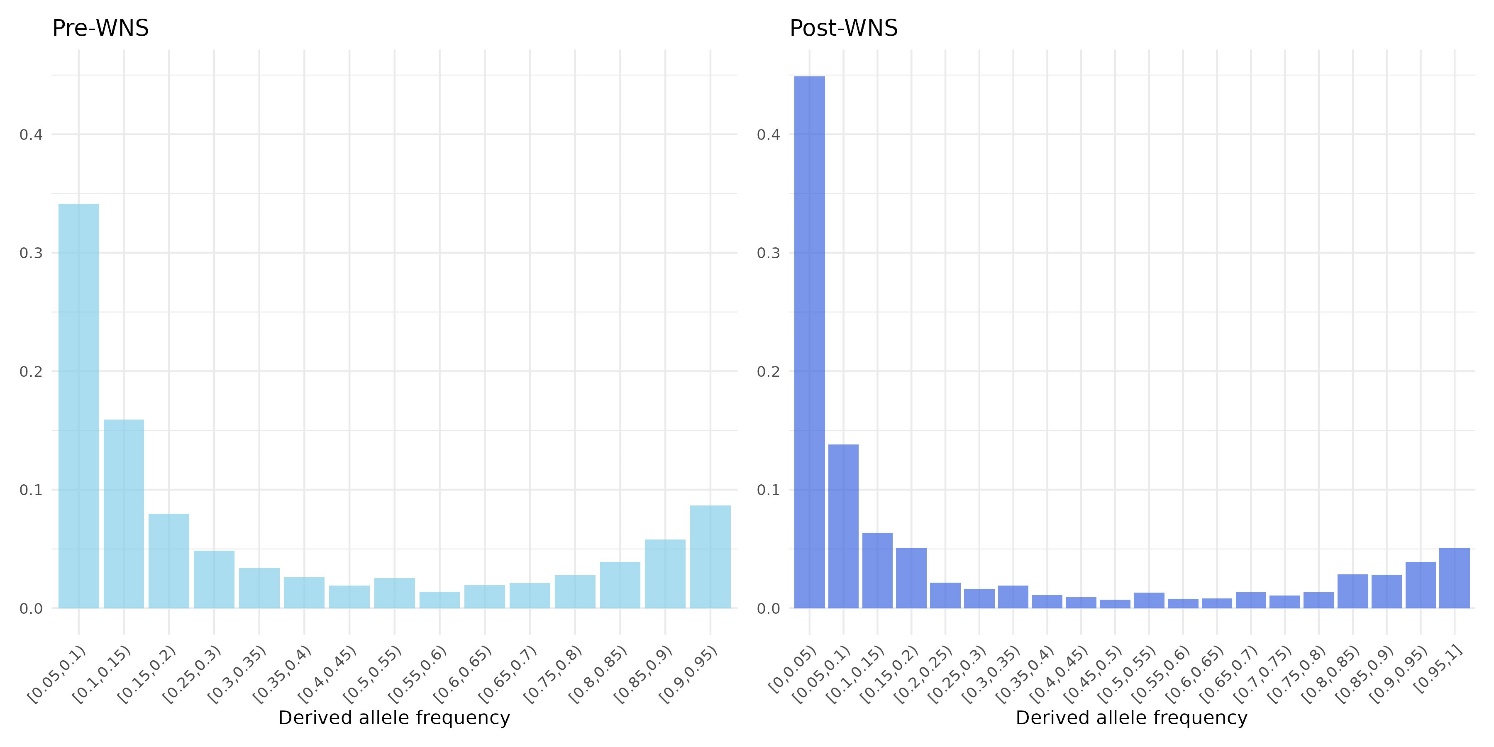


**Arkansas**


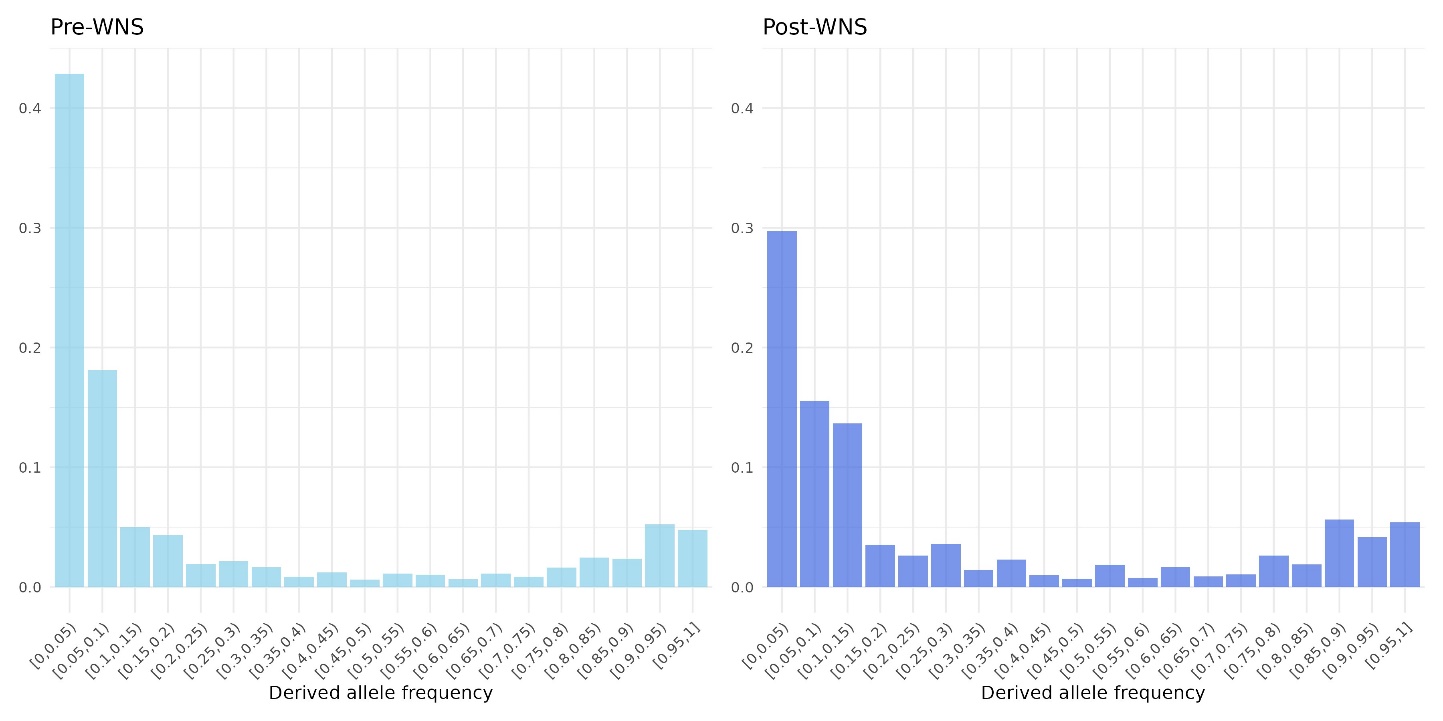


**Missouri**

**New Jersey**


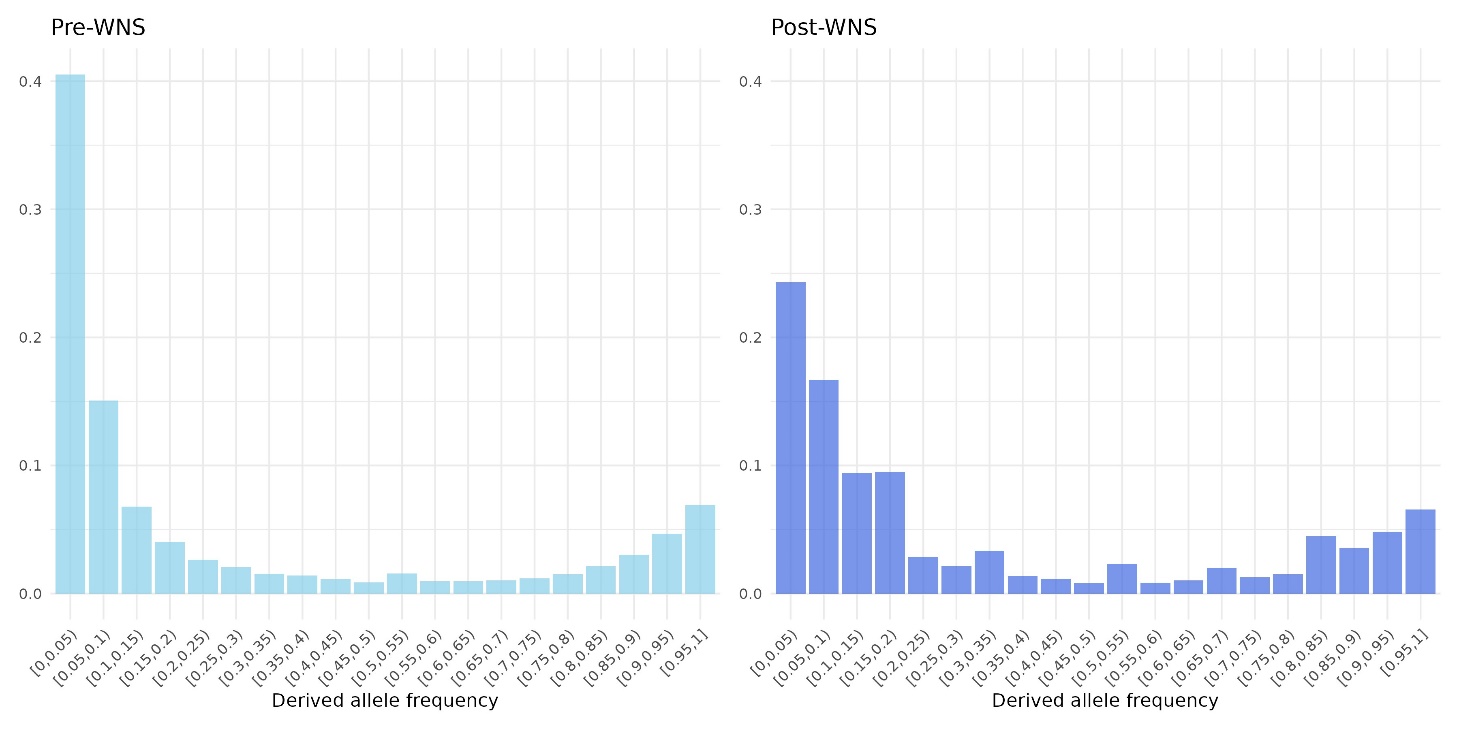


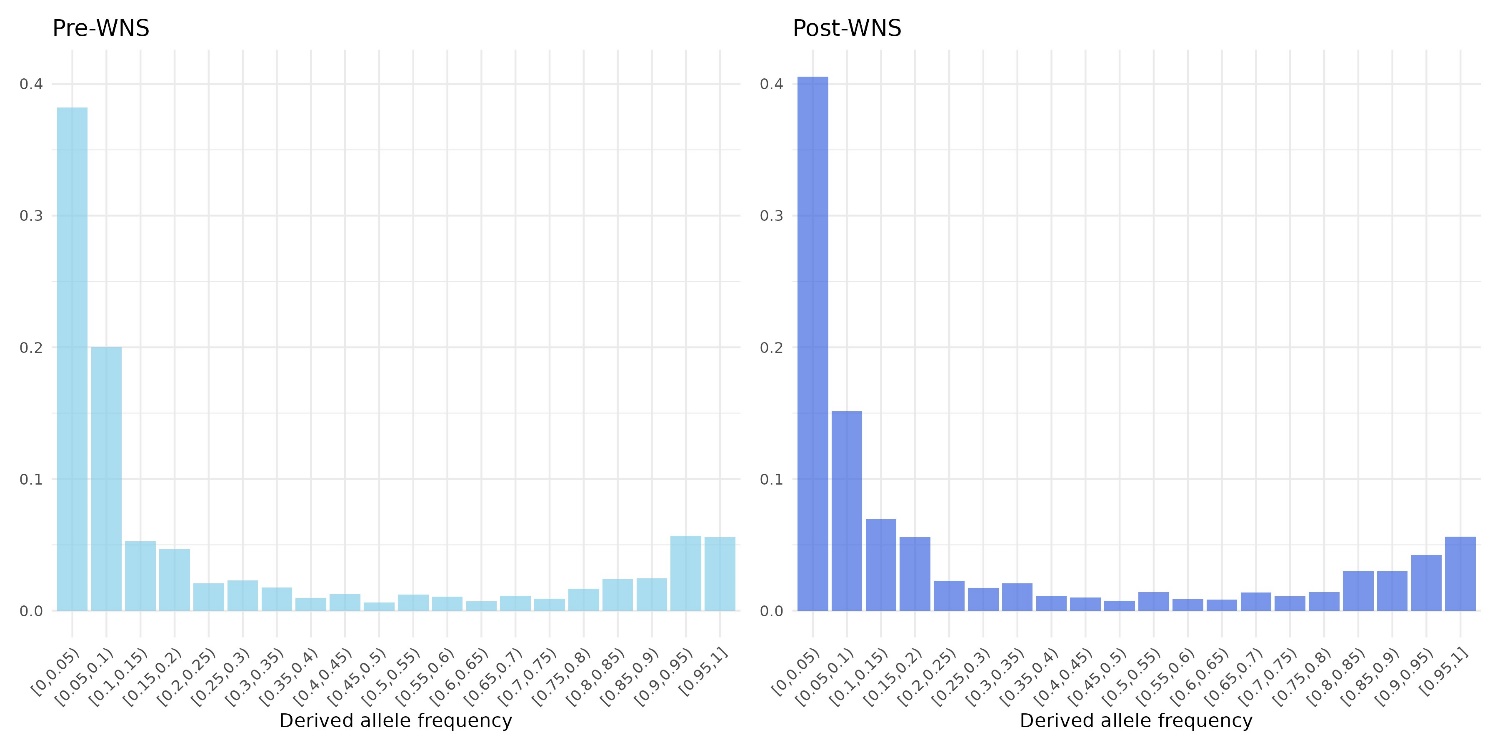


**New York**

**Supplementary Figure 3**. Site frequency spectrum plots for all samples in each time period. A) Pre-WNS samples and B) Post-WNS samples. The y-axis is the frequency of occurrence and the x-axis is the binned allele counts. These graphs were created from the data set employing a 25% missingness threshold.


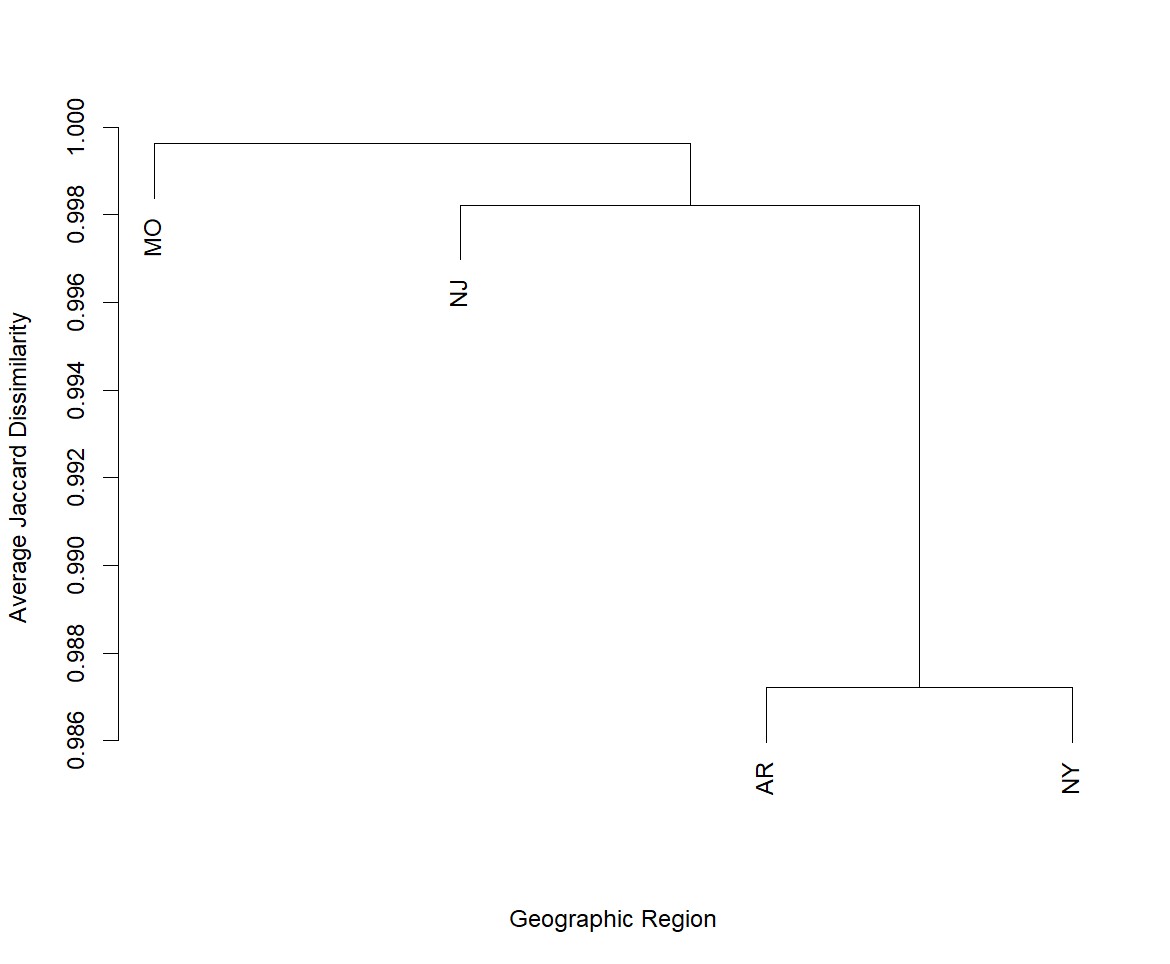


**Supplementary Figure 4**. A dendrogram derived from the average Jaccard dissimilarity between each analysis of each state. The height represents the value of Jaccard dissimilarity.

Rebhan, M., et al. (1998). "GeneCards: a novel functional genomics compendium with automated data mining and query reformulation support." Bioinformatics (Oxford, England) **14**(8): 656-664.
